# Complex Sociality of Wild Chimpanzees Can Emerge from Laterality of Manual Gestures

**DOI:** 10.1007/s12110-019-09347-3

**Published:** 2019-06-24

**Authors:** Anna Ilona Roberts, Lindsay Murray, Sam George Bradley Roberts

**Affiliations:** 1 Department of Psychology, University of Chester, Parkgate Road, Chester, CH1 4BJ UK; 2 School of Natural Sciences and Psychology, Liverpool John Moores University, Byrom Street, Liverpool, L3 3AF UK

**Keywords:** Social networks, Laterality, Gestures, Chimpanzees, Communication

## Abstract

Humans are strongly lateralized for manual gestures at both individual and population levels. In contrast, the laterality bias in primates is less strong, leading some to suggest that lateralization evolved after the *Pan* and *Homo* lineages diverged. However, laterality in humans is also context-dependent, suggesting that observed differences in lateralization between primates and humans may be related to external factors such as the complexity of the social environment. Here we address this question in wild chimpanzees and examine the extent to which the laterality of manual gestures is associated with social complexity. Right-handed gestures were more strongly associated with goal-directed communication such as repair through elaboration in response to communication failure than left-handed gestures. Right-handed gestures occurred in evolutionarily urgent contexts such as in interactions with central individuals in the network, including grooming reciprocity and mating, whereas left-handed gestures occurred in less-urgent contexts, such as travel and play. Right-handed gestures occurred in smaller parties and in the absence of social competition relative to left-handed gestures. Right-handed gestures increased the rate of activities indicating high physiological arousal in the recipient, whereas left-handed gestures reduced it. This shows that right- and left-handed gestures differ in cognitive and social complexity, with right-handed gestures facilitating more complex interactions in simpler social settings, whereas left-handed gestures facilitate more rewarding interactions in complex social settings. Differences in laterality between other primates and humans are likely to be driven by differences in the complexity of both the cognitive skills underpinning social interactions and the social environment.

**Electronic supplementary material:**

The online version of this article (10.1007/s12110-019-09347-3) contains supplementary material, which is available to authorized users.

Laterality, referring to the dominance of one hemisphere of the brain for control of functions or actions, is important for our understanding of human evolution because of the implication that hemispheric specialization may have evolved over time to increase neural capacity and efficiency (Fitch and Braccini 2013; Forrester et al. 2013; Vallortigara and Rogers 2005), and that lateralized individuals should therefore have increased fitness by virtue of being able to multitask. However, McGrew and Marchant (1997a) argue that an individual with a strong lateral bias would likely be disadvantaged relative to its peers because the symmetry of the physical world does not provide that all food or all predators, for example, are present on one side only. In humans, spoken language and manual gestures, defined as voluntary movements of the hands, emerge together and develop alongside each other (Bates and Dick 2002), are linked to left hemisphere specialization (Geschwind 1974), and can both suffer when the brain is damaged, for example, through a stroke (Foundas et al. 1995).

Exploration of this duality was once eagerly pursued in other species, with speculation that handedness and language are closely linked in evolution; as more evidence emerges of distinct species-specific lateralization patterns, however, this link is arguably weakening (Fitch and Braccini 2013). Comparative evidence shows that the vocal repertoire has increased over evolutionary time in those primate species living in more complex large groups, alongside associated increases in the amount of time spent grooming (McComb and Semple 2005) and affiliative gestural communication (Maestripieri 2005). Field studies of primate gestural communication in the wild further demonstrate that manual gestures increase the efficiency of information transfer, and this in turn is important in regulating social dynamics (Roberts and Roberts 2019). If lateral asymmetries in manual gestures are assumed to be manifestations of hemispheric specializations which have evolved over time, it is important to find evidence that lateralized individuals are indeed advantaged. Chimpanzees are our closest living relatives (McGrew 2010) and show complex use of manual gestures and complex sociality in many different contexts (Pollick and de Waal 2007; Roberts et al. 2012a, 2014a). Examining how the laterality of manual gestures is associated with complexity of social behavior among chimpanzees is therefore important to our understanding of the evolution of language and of sociality (Fitch and Zuberbühler 2013).

Among nonhuman primates, more global trends of evidence exist for laterality: for example, for left-handed reaching preferences among several species (MacNeilage 1987), for right-handed biases in chimpanzees and gorillas, and for left-hand bias in orangutans (Hopkins et al. 2012; Marchant and McGrew 1996; McGrew and Marchant 1997a, 1997b, 2001; Prieur et al. 2016; Sanz et al. 2016). Handedness research with chimpanzees has found that, whereas individual biases in hand preference are usually observed, population-level biases are more rarely found (Lonsdorf and Hopkins 2005) and nothing approaching the 90% species-level right bias seen in humans (Faurie et al. 2005; Gilbert and Wysocki 1992). These findings have led to suggestions that although the antecedents of lateralization of function in hand preference were likely present before the *Pan* and *Homo* lineages diverged (Lonsdorf and Hopkins 2005), species-level handedness evolved after and consequently is identified as a strong driver of human evolution (Fitch and Braccini 2013; Harrison and Nystrom 2008). However, emerging evidence supports the view that most lateral biases are socially dependent, the preference being influenced by factors including the context and emotional valence (Fitch and Braccini 2013). In humans, for instance, the semantic content of the message can influence the choice of right or left hand in both co-speech gestures and in gesturing without speaking (Lausberg and Kita 2003). Further, societal learning or cultural norms have been shown to bias laterality of behaviors in humans such as head turning (Karim et al. 2017) or embracing (Packheiser et al. 2019), suggesting that laterality is not genetically fixed but flexible.

Gestural communication shows intentionality and has a learning component (Call and Tomasello 2007; Roberts and Roberts 2017), arguably conferring higher-level strategic attributions than more automatic, albeit informative (Slocombe and Zuberbuhler 2005), species-typical vocalizations. Chimpanzees also make other sounds with their mouth, including lip smacks and buzzes, accompanied by facial asymmetries biased to the right side (Reynolds Losin et al. 2008). These are argued to be under cortical control as opposed to laryngeal phonated vocalizations, including pant-hoots, controlled by the midbrain, which are accompanied by left-side facial biases (Fernández-Carriba et al. 2002). Recent evidence shows that the complex cognitive skills underpinning the use of gestural communication may enable primates to maintain more complex social relationships. For instance, sociality in chimpanzees is associated with specific characteristics of gestural communication, including a large repertoire (Roberts et al. 2019), multimodality (Roberts and Roberts 2019), intentional use (Roberts and Roberts 2018, 2019), and repertoire homogenization (Roberts and Roberts 2017). Given these findings, it is important to explore whether the laterality of manual gestures is related to the strategies that primates adopt to maintain social complexity.

Group size has traditionally been taken as a correlate for social complexity because the number of dyads and triads of social relationships that have to be socially managed increases as a power function of the number of individuals in a group (Aiello and Dunbar 1993; Kudo and Dunbar 2001). However, it is a relatively crude measure of social complexity, and it does not provide a detailed explanation of the challenges that individuals face in fission-fusion social systems, in which changes in the size and composition of subgroups or “parties” occur as a function of the level of stresses incurred by group living. Thus, for primates living in fission-fusion societies, the party size should also be considered as an indicator of the complexity of the social systems (Roberts 2018).

The social system can emerge through the coordination of joint activities (e.g., traveling, resting, or grooming) at the level of the dyad and the group because these micro- and macro-level interactions contribute to the overall proximity that constitutes the nature of the social system (Hinde 1976). “Joint activity” refers to the coordination of behavior between two or more individuals. In particular, coordination of joint activities through gestural communication—whereby signalers direct movement and attention of the recipient toward the joint goal that could not be accomplished individually—can provide a window to understanding the link between social and communicative complexity (Golinkoff 1986, 1993). Signalers use gestures to coordinate behavior intentionally, suggesting that signalers make informed choices on the basis of understanding the comprehension states of others (Tomasello and Vaish 2013). In intentional gestural communication, the signaler has a goal, and if the recipient miscomprehends the goal of the signaler, the latter persists in gesturing by use of informative gestures that refer to the role of the recipient in attaining the desired goal (Tomasello et al. 1985). For instance, the signaler indicates through the gesture what the recipient should do, and if the recipient does not produce the response that matches the goal of the signaler as conveyed by the gesture, the signaler elaborates by using a new gesture enabling comprehension and joint activity to be coordinated (e.g., changing the joint behavior from grooming to joint travel) (Golinkoff 1986, 1993).

Alternatively, joint activity can be achieved spontaneously whereby the salient emotional expression (e.g., pant-hoot) causes a response. This type of communication may be less cognitively complex because the signaler can influence the behavior of the recipient without assessing their comprehension. Spoor and Kelly (2004) argued that this type of coordination may be elicited by a stimulus that can facilitate emotional convergence between partners. For instance, communication can function as a social bonding mechanism that can reduce the stress of the recipient by releasing social neurohormones, and this positive experience facilitates coordination between dyads. Glucocorticoid (GC) is a hormone released in response to elevated levels of arousal. Increased levels of GC can result in displacement behaviors such as scratching, which helps primates cope with stress (Diezinger and Anderson 1986; Schino et al. 1996). Scratching provides one way of indirectly evaluating how external events influence stress states in the recipient and how in turn this is reflected in coordination of joint activity.

In this study, we examine the association between laterality in communicative, manual gestures and social complexity in a community of wild chimpanzees (*Pan troglodytes schweinfurthii*) living in the Budongo Forest, Uganda. Chimpanzees are an ideal species to explore this association because they live in a complex, fission-fusion society whereby the larger community is composed of subgroups or parties that change in composition and duration (Aureli et al. 2008). Most individuals maintain some degree of proximity with all others in their community. In addition, chimpanzees have a differentiated set of social relationships based on grooming. Grooming releases endorphins, an opiate hormone that promotes feelings of relaxation in the recipient, and this positive experience forms the basis for development of social bonds between the partners (Keverne et al. 1989). In particular, unidirectional grooming (in which one chimpanzee grooms another) has an important social bonding value because it reduces stress to a greater degree than mutual grooming (wherein both chimpanzees groom each other at the same time). As a consequence of the bond established through unidirectional grooming, primates show a greater propensity to engage in coordinated activities (traveling, resting, visual monitoring, mutual grooming or co-feeding) and reduced aggression, indicating greater tolerance of the partner. However, the limits of neocortical processing would prevent grooming with every group member because the time required to maintain them would be too expensive (Dunbar 1998). How chimpanzees develop and maintain social relationships is thus a key question because different types of communication are employed for them (Roberts and Roberts 2016a, 2016b). For instance, chimpanzees maintain bonded relationships based on grooming through cognitively complex communication more effectively than through less-cognitively-complex communication (Roberts 2018; Roberts and Roberts 2019).

To examine the link between laterality and sociality we use social network analysis and general linear modeling. In social network analysis, individuals are represented by the nodes (e.g., a chimpanzee) and the social interactions are represented by the edge or a “tie” (e.g., duration of time spent traveling, per hour spent, within 10 m). Whereas statistical methods such as general linear modeling usually focus on examining individual variation in behavior, social network analysis examines variation in behavior between dyads (Croft et al. 2008). Thus, social network analysis can determine how the rates of communication directed toward dyad partners are associated with the duration of time dyads engage in different types of social behaviors. Social network analysis is therefore a valuable tool in determining the factors that influence the social structure since the different types of social relationships and communication are the building blocks for the social system. Table 1 gives summary of key predictions.

**Table 1:** Key predictions about function of laterality

Prediction	Right-handed gesture	Left-handed gesture	Observed
Type of response to the gesture by recipient	Activity change	Emotional display	yes
Communicative repair	Present	Absent	yes
Reduction in displacement activity following the gesture	Absent	Present	yes
Association with greater morphological complexity	Absent	Present	yes
Party size	Small	Large	yes
Association with evolutionarily urgent contexts	Present	Absent	yes

First, we test the hypothesis that laterality of the gestures will be differentiated by context. Studies in humans indicate that targeted reaching movements toward conspecifics, objects (Mutha et al. 2010), or tools (Sunderland et al. 2013) are controlled by the left hemisphere; hence, right-handed gestures may facilitate the signaler’s accuracy of movement, yielding important fitness advantages by improving social coordination. For example, in evolutionarily urgent contexts, such as mating or mating deception, right-handed gestures can improve social coordination by precisely indicating the recipient of the gesture (see Video 1, available online, for an example). In social bonding contexts such as unidirectional grooming, a chimpanzee’s ability to induce a conspecific to move a body part to facilitate grooming may rely on clearly indicating the specific body part the recipient should move (see Video 2, available online, for an example). By accurately indicating the target of gesturing, chimpanzees may increase gesture comprehension, and this in turn may increase the likelihood of eliciting the appropriate response and reciprocity of grooming. Thus, relative to left-handed gestures, right-handed gestures may increase the efficiency of social coordination in goal-directed contexts. In this case we would expect a positive association between the rate of right-handed gestures and (1) the duration of social interaction, (2) response, and (3) reciprocity.


(AVI 25607 kb)



(MPG 11958 kb)


Left-handed gestures may have an adaptive function by influencing the behavior of the recipient (Spoor and Kelly 2004). Human right-hemisphere-controlled communication is more expressive than left-hemisphere-controlled communication (Sackeim et al. 1978). Right-hemisphere-controlled communicative complexity can induce compatible affect in recipients, and this can facilitate social coordination between two interacting individuals (Owren and Rendall 2001). Communicative complexity underpinning the use of left-handed gestures can facilitate social coordination through emotional convergence. In this case, communication functions as a social bonding mechanism that facilities social coordination (e.g., joint travel). Thus, left-handed gestures may facilitate social coordination in non-goal-directed contexts more effectively than right-handed gestures, and this will be expressed as a positive correlation between social coordination and use of left-handed gesture.

Second, we hypothesize that characteristics of the audience will influence laterality of gesturing. Manual gestures can accurately reorient the attention of the recipient by making a definite reference to the goal so that it becomes the shared focus of attention between signaler and recipient. Manual gestures used in coordination contexts are visual (received through seeing behavior, e.g., begging with the hand), tactile (received through tactile sensation, e.g., gentle touch) and auditory (e.g., using own body as in hand clap, or objects such as shaking a branch with a hand to make a sound). Although flexible meaning-making can develop through tactile gestures (Bard et al. 2017) and auditory gestures (Matsumoto-Oda and Tomonaga 2005), visual gestures have many different forms (Roberts et al. 2012b) and occur in the same form through many different contexts (Pollick and de Waal 2007; Roberts et al. 2012a, 2013, 2014a). Visual gestures are directed at the entity that is seen as farther away from the gesturing hand. Further, the gesture can disambiguate something that the recipient sees, rather than feels or hears, as the target of the gesture (Rolfe 1996). Whereas tactile or auditory gestures can influence the recipient through physical or auditory contact, visual gestures require an understanding that the recipient can be causally influenced by distal means (Camaioni 1993).

These conditions create different cognitive requirements for communication through visual gestures relative to tactile or auditory gestures because visual gestures require the recipient to monitor the channel of communication and the signaler to integrate shared attention of the recipient to communication and to the goals in deciding how to communicate (Camaioni 1993). Thus, visual gestures can be argued to be underpinned by higher cognitive complexity than tactile or auditory gestures. For instance, chimpanzees frequently use visual gestures to communicate in grooming contexts but elaborate on them through tactile or auditory gestures, when visual gestures have been unsuccessful (Roberts et al. 2013). In children on the autism spectrum, who exhibit difficulty in engaging in mutual attention, caregivers are less likely to depend solely on conventional communication means, often combining them with physical actions that increase the perceptual salience of referents to draw the child’s attention (Adamson and Bakeman 1991). The nature of the cognitive demands underpinning the use of visual gestures when there is a need for increased joint attention may interact with the influence of social audiences on the recipient’s behavior. Such sensitivity to the presence of others is important for managing social relationships. This is particularly the case for primates, among whom conspecifics may influence one’s position in the group and one’s fitness. Studies with children on the autism spectrum show impaired performance in comparison with control groups on joint attention tasks, when a social audience is present (Chevallier et al. 2014). This is further supported by evidence across a wide range of species, including cockroaches, rats, monkeys, and humans, which shows that the presence of conspecifics reduces the complexity of behavior by increasing arousal (Zajonc and Sales 1966). Increase in arousal makes simple tasks easier but more complex tasks, such as engagement in mutual attention, harder. Arousal may increase in the presence of an audience of partners who are similar in age to oneself because these partners are more likely to be socially important and visually engaged with the signaler (Leary and Allen 2011; Roberts and Roberts 2017). In larger audiences, the need to integrate information from numerous sources is cognitively demanding, increasing arousal and possibly constraining the complexity of intentional communication. Based on these findings we predict that visual gestures will be more common in smaller parties than tactile or auditory gestures.

The presence of an audience can result in higher arousal but also activate greater skills of voluntary control (Hamilton and Lind 2016). For instance, when the visual attention of the dominant social bond partner of an estrous female chimpanzee is directed at a subordinate male initiating mating, chimpanzees use more-intense auditory gestures, but when this visual attention is directed away, chimpanzees use less-intense visual gestures (Roberts and Roberts 2015). This finding suggests that audience effects in mating contexts may be a consequence of the ability to modify gestures due to being watched by the dominant male. Thus, if left-handed gestures are simply an arousal response to the presence of conspecifics, then they will occur in response to the presence of an audience of similar age as the signaler. Alternatively, if the use of left-handed gestures is flexibly tailored to the recipient, then they will be used when same-age partners as the recipient are present in the audience.

Third, we hypothesize that the strength of the social bonds with the partner (as shown by degree of mutual grooming) will influence the laterality of the gesture. One key question in communication studies involves the selection pressures behind communication design and function. The degree of conspicuousness in communication may reflect an adaptive process for increasing the efficiency of information transfer (Dawkins and Guilford 1997). Emotional displays are widespread in the animal kingdom in evolutionarily urgent contexts to provide salient emotional information (Forrester and Todd 2018; Mendl et al. 2010). However, in frequent one-to-one interaction, low-intensity communication has adaptive value over high-intensity, salient emotional expression (Roberts and Roberts 2016a, 2016b). Low-intensity communication relative to high-intensity communication has important fitness benefits by reducing stress and positively influencing well-being and health in frequent one-to-one interactions (Roberts and Roberts 2016a, 2016b). For example, chimpanzees use a higher rate of low-intensity visual gestures, as compared with higher-intensity tactile or auditory gestures, toward partners with whom they spend a longer duration of time in activities such as mutual grooming, joint travel, and joint attention. However, low intensity of a display is inconspicuous, and this may impose a limit on fast detection and interpretation of these gestures (Roberts and Roberts 2016a).

It has been argued that in cooperative contexts such as grooming, where it is in the interests of the signaler to signal and of the recipient to respond, there should be selection for the recipient to become sensitive to the signaler and thus for the signal to become inconspicuous but still effective at influencing the recipient (Dawkins and Guilford 1997). An example would be the single, visual, left-handed pointing between human mother-infant dyads versus single, visual, right-handed pointing between unrelated dyads (Butterworth 2003). Whereas inconspicuous, low-intensity emotional communication may be effective in communication between strongly bonded partners, it may be insufficiently salient to enable effective coordination between individuals with whom social bonds are weaker. Thus, the increased informational value of right-handed gestures in contexts of low-intensity signaling may have evolved as an adaptation that facilitated more effective information transfer relative to left-handed gestures when social bonds are weaker. Here we predict that, when social bonds are weaker, chimpanzees will engage in social interactions through right-handed gestures more effectively than through left-handed gestures.

## Methods

Six adult males and six adult females from the Sonso community of East African chimpanzees (*Pan troglodytes schweinfurthii*) were observed at the Budongo Conservation Field Station in Uganda. The demographic details of each focal subject and observation duration for each focal subject are given in ESM-1 (Table S1), alongside descriptive statistics for all variables entered into the models (ESM-1, Tables S2–S3). The details of the site, subjects, data collection, video analysis, and classification of the gestures have been described previously (Roberts et al. 2012a, 2012b, 2013, 2014a; Roberts and Roberts 2015) In total, we examined 545 bouts of adult-to-adult communication (1044 instances of gestures). We used quantitative focal animal follows, choosing subjects systematically and recording the focal subject’s behavior during a standardized observation period of 18 min such that consecutive samples of the same focal subject were taken at least 20 min apart. We aimed to sample subjects’ behavior equally at different times of the day and study period, at least once during a week. However, the observation duration varies between individuals because the fission-fusion social system of chimpanzees in the wild means that individuals are not encountered at similar rates. Thus, although we aimed to sample each of the individuals equally during the study period, this was not always possible given the available resources and other fieldwork constraints.

The 18-min focal follows consisted of 9 scans at 2-min intervals of the identity of the individuals present within 10 m of the focal subject and those who were more than 10 m away, but who were in the same party. The party was defined as the group of individuals within a spread of around 35 m. Moreover, the distance to nearest adult neighbor, their activity (e.g., feeding, resting, travel, and whether the focal subject was the recipient of the behavior if applicable for directed behaviors such as grooming), and bodily orientation relative to focal subject were recorded. The activity of the focal subject was also recorded so that it was possible to determine whether both the focal subject and the nearest neighbor performed the same activity at the same time (e.g., joint resting). The scan sampling of social behavior listed above at 2-min intervals was accompanied by continuous recording of all instances of mating. Further, continuous videotaping of chimpanzee gestural communication occurred along with verbal description of the context (e.g., the identity of the signaler/recipient, their behavior prior to and after production of the gesture, goal-directedness, distance and bodily orientation between signaler and the recipient).

### Identifying Gestures from Video Footage

The video footage was viewed on a television and coded. Nonverbal behaviors were coded for a number of morphological features which formed the basis for objective judgment of the similarity in morphology (i.e., presence/absence and type of head, trunk, arm movement; posture; social orientation) (Roberts et al. 2012b). For intentionality, the details of this classification can be found in previous literature (i.e., Roberts et al. 2014a). Further, the details of association of each gesture type with each of these criteria have been given in Roberts and Roberts (2018). We included criteria such as bodily orientation and persistence in communication to identify intentionality of the gestures. Persistence was identified when the signaler stopped gesturing after the recipient responded to the gesture or modified the type of communication when the recipient failed to respond (i.e., Roberts et al. 2014a). Furthermore, intentionality was assessed on the basis of bodily/visual orientation between the signaler and the recipient. Our findings show that the mean percentage ± SD [95% CI] of cases of all gesture types associated with the presence of bodily orientation by the signaler toward the recipient during production of the gesture was 91.5 ± 18.5%, [87, 95]. Moreover, the mean percentage ± SD [95% CI] of cases of all gesture types associated with the presence of recipients’ bodily orientation toward the signaler, when the signaler’s bodily orientation towards the recipient was absent, was 6.9 ± 15.4% [3, 10]. Finally, the mean percentage ± SD [95% CI] of cases of all gesture types where neither the signaler nor the recipient were bodily oriented toward one another during production of the gesture was 1.5 ± 11% [0, 3]. Thus, almost all of the gestures in our dataset were intentional, in accordance with previously established criteria for defining intentionality in preverbal humans and primates (Bard 1992).

Gestures occur as single events or in sequences, defined as one (or more than one) gesture made consecutively by one individual, toward the same recipient, with the same goal, within the same context, within a maximum of 30 s interval. For each single gesture or sequence we noted the identity of the signaler (the individual performing a gesture); the identity of the recipient (the individual at whom the gesture was most clearly directed, as determined from the orientation of head and body of the signaler during or immediately after performing a gesture—i.e., the signaler had the recipient within its field of view) and the context (see Roberts and Roberts 2016b for ethogram). Gestures were classified according to the modality following the ethogram detailed elsewhere (Roberts and Roberts 2016a, 2016b). Only those gesture events where the recipient was within 10 m of the signaler during the gesture production were considered, taking into account the ability of the recipient to perceive the gesture. In this study we did not categorize chimpanzees as right-handers or left-handers. All measures of communication in this study were based on rates (e.g., frequency of left-handed and right-handed gestures produced toward the recipient per hour spent within 10 m of the recipient), duration of social behavior when signaler and recipient were nearest neighbors and were within 2 m of each other per hour spent in same party) as well as rates (e.g., scratch produced or received or copulations per hour spent within 10 m of the recipient). Further, we derived bouts of unidirectional grooming from video footage, whereby the focal subject but not the nonfocal subject was providing grooming to the recipient. If the nonfocal subject returned the grooming by either mutually grooming with the focal subject or unidirectionally grooming them within 2 min of grooming cessation by the focal subject, this unidirectional grooming bout by the focal subject was categorized as reciprocated. In total, 578 min of unidirectional grooming was recorded in the study period, with the data taken for unidirectional grooming between adult individuals only.

In GLMM analysis only independent bouts were considered—that is, only those bouts (containing one or several gestures) where only one laterality of manual gesture was present (either left or right). In social network analyses we took into account frequencies of all right-handed and left-handed gestures when the recipient was within 10 m of the signaler, calculating their rate per hour spent within 10 m (Hopkins et al. 2001).

In order to ensure that the sampling procedure did not bias our results, we tested similarity in association patterns. The details of these analyses were outlined in detail previously (Roberts and Roberts 2016a). The laterality measure was determined as follows.

### The Dyadic Laterality of Gesture Measure

The dyadic laterality of gesture measure (DL) is the rate at which focal subject A communicated with right or left hand to nonfocal subject B when B was within 10 m of focal subject A, per hour spent within 10 m of the nonfocal subject B, or

$$ {\mathrm{DL}}_{\mathrm{AB}}=\left({\mathrm{L}}_{\mathrm{AB}}\ast 60\right)/\mathrm{P}{10}_{\mathrm{AB}}\ast 2 $$

where L_AB_ = the number of times A communicated with B when in close proximity (within 10 m) and the communication was right- or left-handed; P10_AB_ = the number of times A was in close proximity (within 10 m) of B; 2 = duration of instantaneous subsample interval in minutes; and 60 = the number of minutes in an hour.

The chimpanzee dyads were described in terms of kinship similarity, sex similarity, reproductive similarity, and age proximity following previous studies (Mitani 2009). The details of this categorization of attribute data can be found in (Roberts and Roberts 2016b).

### Inter-Observer Reliability

An experienced field assistant, unaware of the aims of the study, recorded behavior and social behavior patterns. The field assistants annually undergo an inter-observer reliability test, the interval deemed to be sufficient to maintain the consistency of scoring of the group composition and proximity and activity across field assistants, with results consistently above 0.85 (Spearman’s rank correlation coefficient, *r*_s_). The filming of gestures and context commentary was carried out by AR, ensuring independence of data collection of social behavior and of the gestures. The coding was validated by the second coder, who scored a random sample of 45 gesture sequences, coding the function and modality of the gestures, assigning them correctly to each of the categories. Cohen’s kappa coefficient showed that reliability was excellent for function (κ = 0.70) and modality of gesturing (κ = 0.946) (Bakeman and Gottman 1997). Another sample of 50 sequences of gestures was coded by a second coder for intentionality (response waiting and persistence), and Cohen’s kappa coefficient indicated good reliability (κ = 0.74) (Bakeman and Gottman 1997).

## Analyses

### Generalized Linear Model (GLMM)

We used Generalized Linear Mixed Models (GLMM) to examine the factors influencing the binary response variables, such as presence of a left-handed or right-handed gesture. Only significant findings are reported in the Results section; the details of all GLMM models are shown in ESM-2, Tables S1–S14. To avoid correlation between variables, we determined Variance Inflation Factors (VIF) from a model that included the fixed effects using a linear regression model. There was a high colinearity for proximity and visual attention since these variables overlapped in time with other variables (VIF > 10), so proximity and visual attention were not included in GLMM models. Other variables showed no colinearity. The data in these GLMMs were hierarchically structured—Level 1 was the focal individual and Level 2 was the recipient of the gesture. These models represent a form of a regression where the data has a hierarchical clustering structure. The models were fitted using a binomial error structure with logit link. The random effects included were the focal individual identity and the focal individual identity by recipient identity—for these effects random intercepts were used. All analyses were carried out using IBM SPSS Statistics 22.

### Social Network Analysis

The gesture networks were directed and weighted. Each cell in the matrix had a continuous value representing the behavior, rather than a 1 or a 0 indicating the presence or absence of a tie. From these network matrices, centrality measures were calculated using normalized degree centrality (Croft et al. 2008). Normalized degree centrality is the average value of each row or column of the network matrix (i.e., the average value of that behavior for each focal chimpanzee). Since in all instances in the communication networks were directed (i.e., the rate of right-handed gesture networks) the in-degree and out-degree were calculated separately. Out-degree refers to behaviors directed by the focal chimpanzee to conspecifics; in-degree refers to behaviors directed by conspecifics toward the focal chimpanzee. Multiple Regression Quadratic Assignment Procedure (MRQAP) regression was used to determine the relationships between sociality and communication networks (Borgatti et al. 2013). The number of permutations used in this analysis was 2000. For the node-level regressions, we used a similar procedure, using 10,000 random permutations to assess the effect of a number of predictor variables (e.g., the out-degree for laterality of gestures, sex of focal chimpanzee) on the outcome variable (e.g., proximity in degree). Finally, to examine correlation between attribute data (e.g., the total duration of observation) and network data (e.g., right-handed gesture network), the Geary’s C statistic was used. When there is no association between variables, the Geary statistic has a value of 1.0, with values of less than 1.0 indicating a positive association and values over 1.0 indicating negative association. UCINET 6 for Windows was used to carry out all data transformations and analyses (Borgatti et al. 2014). Only significant findings are reported below in the Results section; the details of all models are shown in ESM-2 Tables S15–S18. The results are summarized in visual form in ESM-3 through ESM-6.

### Sampling Duration

In this study we used MRQAP, node-level regression, and GLMM to examine an average of 12.52 (range 8.33–18.63) hours of independent focal data per individual subject. ESM-1 Table S4 provides details of the analyses of the relationship between the total duration of observation and each of the laterality networks, showing that there was a sufficient sampling duration.

## Results

### Rates of Right- and Left-Handed Gestures

The mean rates (overall range) of right- and left-handed gestures per hour spent within 10 m of the recipient were 0.18 (0–3.53) and 0.41 (0–22.9), respectively. The mean normalized degree (the percentage of potential connections chimpanzees had with others) (overall range) for right- and left-handed gestures was 25 (9–73) and 28 (0–64), respectively. Table 2 presents the categorization of manual gestures according to laterality, modality, and type.

**Table 2:** Manual gestures according to laterality, modality and type

Category	Gesture type
Left-handed gestures	
Visual	Unilateral swing, touch self, vertical extend, stretched extend, limp extend, hand bend, forceful extend, arm raise, arm flap,
Auditory short-range	Wipe
Auditory long-range	Shake stationary, hit object, shake mobile, drag object
Tactile	Touch backhand, embrace, grab, pull another, hold hands, touch long, rub, push by hand, stroke short, tickle, tap another, offer hand,
Right-handed gestures	
Visual	Unilateral swing, arm beckon, vertical extend, limp extend, forceful extend, stretched extend, arm flap, stiff extend, retrieve, linear sweep, hand bend, arm raise,
Auditory short-range	Clip by hand, tap object
Auditory long-range	Shake stationary, shake mobile, slap object, knock, hit object, break
Tactile	Push by hand, touch backhand, tap another, shake limb, pull another, poke, offer hand, embrace,

### Laterality of the Manual Gesture Is Associated with Presence of Response by the Recipient

Higher frequency of left-handed gestures in the sequence was associated with presence of overall response to the gesture (β = 0.815 *p* = 0.007). When the dyad partners did not engage in mutual grooming, the response to the gesture was more likely present when signalers used right-handed gestures (β = −0.710, *p* = 0.029). In contrast, when dyad partners engaged in mutual grooming, there was a trend for the response to the gesture to be more likely present when signalers used left-handed gestures (β = 1.284, *p* = 0.077).

### Laterality of the Manual Gesture Is Associated with Type of Response by the Recipient

Response by activity change was more likely than response by communication when the signaler used right-handed gestures (β = 0.647, *p* = 0.031).

### Laterality of Manual Gestures Is Associated with Reciprocity of Grooming Bouts

A grooming bout was more likely to be reciprocated when the dyad partners engaged in mutual grooming (β = −22.606, *p* < 0.001) and when signalers used right-handed gestures (β = −22.759, *p* < 0.001). Right-handed gestures were more likely than left-handed gestures when the reciprocity to unidirectional grooming bout was present (β = −22.273, *p* < 0.001) and when the partners did not engage in mutual grooming (β = 22.788, *p* < 0.001).

### Right-Handed Gestures Predict Presence of Communicative Repair

Communicative repair was more likely than all other types of communication combined when chimpanzees used right-handed gestures (β = −1.325, *p* = 0.004). Further, communicative repair was more likely than all other sequence types of communication combined when the dyad partners produced right-handed gestures (β = −1.210, *p* = 0.008).

### Laterality of Manual Gestures Is Associated with Demography, Audience Characteristics, and Type of Accompanying Behavior

**Fig. 1:**
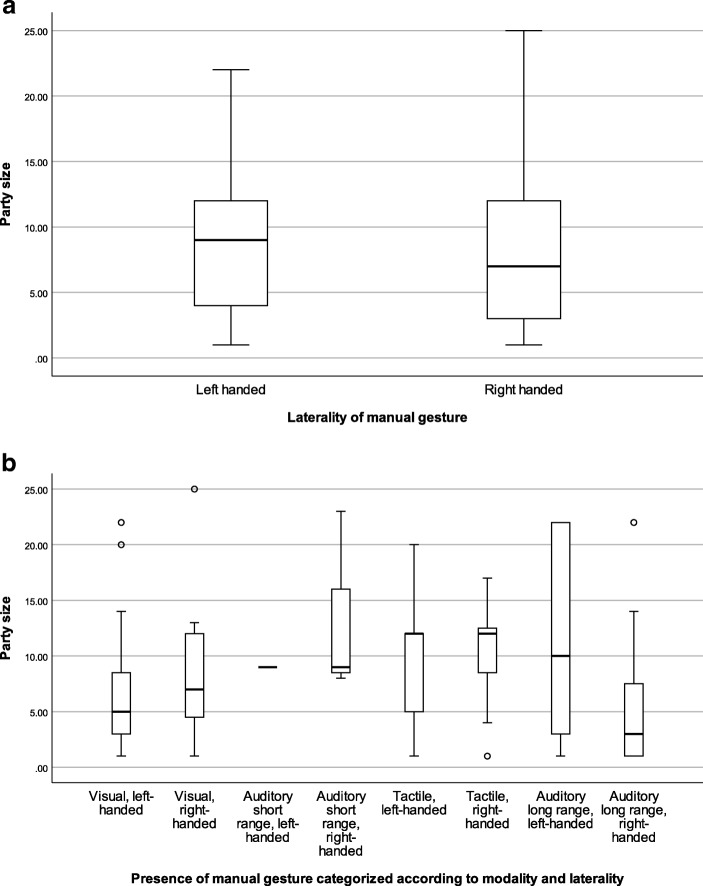
Presence of right- and left-handed gestures by party size: (a) all manual gestures combined and (b) manual gestures categorized according to modality. For illustrative purposes, dyads were classified by the presence or absence of right-handed and left-handed gestural communication

We explored two models. In Model 1, right-handed gestures were more likely than left-handed gestures when the recipient was of the same age (β = −0.986, *p* < 0.001), when signaler and recipient were kin (mother-offspring dyad) (β = −14.578, *p* < 0.001), when the size of the party declined (β = −0.057, *p* = 0.026, Fig. 1a), when the focal subject was groomed longer by the recipient (β = 0.134, *p* = 0.025), when the duration of mutual grooming between focal subject and the recipient was shorter (β = −0.317, *p* = 0.021), when the number of tactile gestures in the sequence declined (β = 0.783, *p* = 0.002), when the rate of lip-smacks in the sequence increased (β = 4.005, *p* < 0.001), and when the synchronized low-intensity pant-hoot (β = 14.066, *p* < 0.001) and the synchronized high-intensity pant-hoot (β = 1.522, *p* = 0.002) were absent. In Model 2, we removed party size from the model, replacing it with the variable denoting the presence or absence of audience members of the same age as the focal subject and the same age as the recipient. When these variables were included the Akaike values decreased from 656.955 (Model 1) to 645.820 (Model 2), indicating a better model fit. The results showed that right-handed gestures were more likely than left-handed gestures when same-age partners of the recipient were absent (β = 0.914, *p* = 0.015).

### Size of the Party Is Differentiated by Repertoire Size and Frequency of Left- and Right-Handed Gestures

Further, the third set of models showed that larger repertoire size of right-handed gestures in the sequence was more likely when party size declined (β = −3.711, *p* = 0.001), when the same age partners as the signaler were absent (β = −0.828, *p* = 0.012) and when same age partners as the recipient were absent (β = −1.605, *p* = 0.006). Finally, when examining the association between party size and frequency of left- and right-handed gestures in the sequence categorized according to modality, we found that there was a higher frequency of visual (β = −2.507, *p* < 0.001) and auditory long-range (β = −4.117, *p* < 0.001) right-handed gestures within the sequence in smaller parties (Fig. 1b).

### Laterality of Manual Gestures Predicts Duration of Time Spent in Social Bonding Behavior within Dyad

**Fig. 2:**
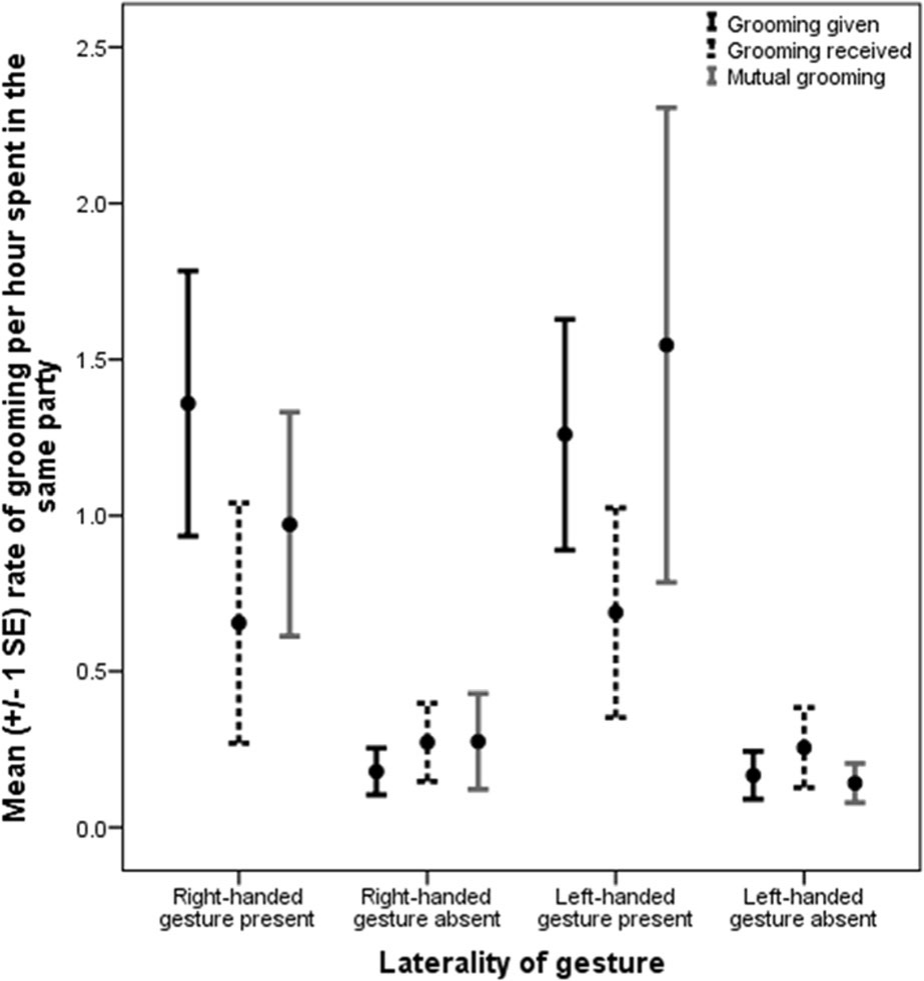
Mean rate of grooming, per hour spent in the same party, by laterality of gestural communication. For illustrative purposes, dyads were classified by the presence or absence of right-handed and left-handed gestural communication

The rate of left-handed gestures was significantly positively associated with longer duration of time spent in joint travel (β = 0.205, *p* = 0.034), mutual grooming (β = 0.241, *p* = 0.026), and attention present (β = 0.204, *p* = 0.025), but negatively associated with copulation rate (β = −0.156, *p* = 0.028) and scratch received rate (β = −0.176, *p* = 0.02; Fig. 2). The rate of right-handed gestures was significantly positively associated with duration of unidirectional grooming (β = 0.278, *p* = 0.015), proximity to 10 m (β = 0.253, *p* = 0.007), copulation rate (β = 0.505, *p* = 0.005), and the rate at which scratching was produced by the non-focal subject in the presence of the focal subject (scratch received, β = 0.196, *p* = 0.044). Duration of feed, rest, groom receive, attention absent, proximity to 2 m, and rate of scratch produced were not associated with laterality. Finally, the rate of lip-smack produced was significantly positively associated with the rate of scratch produced in response (β = 0.182, *p* = 0.040) and significantly negatively associated with the rate of scratch received in response (β = −0.100, *p* = 0.049).

### Laterality of Manual Gestures Produced and Received Predicts Position in the Social Network

Chimpanzees with a high out-degree of mutual resting (β = 1.135, *p* = 0.033), joint travel (β = 0.961, *p* = 0.032), mutual grooming (β = 0.995, *p* = 0.025), grooming received (β =0.938, *p* = 0.049), attention present (β = 0.903, *p* = 0.043), attention absent (β = 1.047, *p* = 0.035), and proximity to 2 m (β = 1.004, *p* = 0.034) had a high in-degree of right-handed gestures. Chimpanzees with a high-out degree of unidirectional grooming had a high out-degree of right-handed gestures (β = 1.161, *p* = 0.032) and low in-degree of left-handed gestures (β = −0.905, *p* = 0.022). Finally, chimpanzees with a high out-degree of mating had a high out-degree of right-handed gestures (β = 1.489, *p* = 0.014) and low in-degree (β = −0.868, *p* = 0.036) and out-degree (β = −0.911, *p* = 0.035) of left-handed gestures. Out-degree of feed and proximity to 10 m was not associated with laterality.

### Laterality of Manual Gestures Predicts the Complexity of Communication within Dyads

Chimpanzees who directed a higher rate of left-handed gestures at the dyad partner also directed a higher rate of gestures representing almost all complexity categories: bodily (β = 0.326, *p* = 0.008), manual (β = 0.810, *p* = 0.001), combined (β = 0.207, *p* = 0.042), non-combined (β = 0.619, *p* = 0.001), objects (β = 0.192, *p* = 0.044), non-objects (β = 0.577, *p* = 0.009), indicative (β = 0.160, *p* = 0.043), non-indicative (β = 0.830, *p* = 0.001), events (β = 0.560, *p* = 0.007), unimodal (β = 0.616, *p* = 0.004), high-amplitude call (β = 0.277, *p* = 0.025), facial expression (β = 0.303, *p* = 0.023), attention present (β = 0.327, *p* = 0.016), attention absent (β = 0.692, *p* = 0.001), homogeneous (β = 0.441, *p* = 0.007), heterogeneous (β = 0.625, *p* = 0.003), single (β = 0.704, *p* = 0.001), rapid (β = 0.327, *p* = 0.016), repetitive (β = 0.500, *p* = 0.003), non-repetitive (β = 0.418, *p* = 0.01), close proximity (β = 0.699, *p* = 0.001), far proximity (β = 0.317, *p* = 0.016), piloerection (β = 0.349, *p* = 0.016), visual (β =0.399, *p* = 0.020), tactile (β = 0.901, *p* = 0.001), auditory short-range (β = 0.113, *p* = 0.049), and repertoire size (β = 0.515, *p* = 0.011), response present (β = 0.683, *p* = 0.009) and response absent (β = 0.372, *p* = 0.010). Chimpanzees who directed a higher rate of right-handed gestures at the partner, directed a higher rate of gestural communication that was manual (β = 0.103, *p* = 0.047), indicative (β = 0.186, *p* = 0.042), low-amplitude call (β = 0.242, *p* = 0.046), penile erection (β = 0.446, *p* = 0.006), heterogeneous (β = 0.137, *p* = 0.043), single (β = 0.116, *p* = 0.043), persistence (β = 0.274, *p* = 0.028), elaboration (β = 0.253, *p* = 0.032), and response absent (β = 0.253, *p* = 0.028). Further, high-amplitude call (β = −0.158, *p* = 0.011) and response present (β = −0.107, *p* = 0.031) were negatively associated with the rate of right-handed gestures. Rate of auditory long-range gestures and repetition was not associated with laterality.

### Laterality Predicts Gesture Function within Dyads

A higher rate of left-handed gestures predicted a higher rate of threat to dominate (β = 0.396, *p* = 0.012), gesture to groom give (β = 0.160, *p* = 0.038), gesture to groom mutual (β = 0.406, *p* = 0.010), gesture to groom receive (β = 0.439, *p* = 0.011), synchronized high-intensity pant-hoot (β = 0.105, *p* = 0.049) and high-intensity pant-hoot solo (β = 0.222, *p* = 0.032). A higher rate of left-handed gestures predicted a lower rate of copulation (β = −0.156, *p* = 0.03) and other threat (β = −0.119, *p* = 0.034). A higher rate of right-handed gestures predicted a higher rate of copulation (β = 0.505, *p* = 0.004), gesture to groom give (β = 0.196, *p* = 0.049), and other threat (β = 0.187, *p* = 0.049). A higher rate of right-handed gestures predicted a lower rate of threat to dominate (β = −0.235, *p* = 0.006), gesture to groom mutual (β = −0.237, *p* = 0.003), and high-intensity pant-hoot solo (β = −0.098, *p* = 0.049). Synchronized low-intensity pant-hoot and greeting were not associated with laterality.

## Discussion

Our findings provide important comparative data on laterality in a wild population of chimpanzees based on natural, spontaneous gestures, and linking them to social dynamics. Whereas previous research on functional laterality in great apes has focused on factors such as rearing history (Hopkins et al. 2004), whether the task is one- or two-handed (Llorente et al. 2009), or whether the target of a manual action is animate or inanimate (Forrester et al. 2011, 2012), here we provide the first systematic evidence for an association between social relationships and the laterality of multimodal gestures in different contexts—specifically, the role of the type of social relationship, the audience, and the size of the network.

Our study provides some previously undocumented evidence that right-handed gestures appear to be more goal-directed than left-handed gestures. Right-handed gestures were indicative and idiosyncratic. The fact that right-handed gestures were used more often than left-handed gestures in communicative repair sequences shows that chimpanzees used right-handed gestures when there was a communication failure between the signaler and the recipient, to improve the efficiency of signaling. Right-handed gestures predicted goal-directed reactions conforming to the goal of the signaler rather than emotional reactions, which were more commonly associated with left-handed gestures. Right-handed gestures appear to effectively coordinate the recipient’s attention and behavior toward a common goal. Use of right-handed gestures in coordination contexts requires the coordination of attention and communication to a goal and to one another, providing evidence that signalers understand others as intentional beings with comprehension states about the goal. Such a capacity to pay simultaneous attention to the social partner and the external goals has been previously shown in language-trained chimpanzees (Roberts et al. 2014b) and captive bonobos (Pika and Zuberbuhler 2008), revealing that skills for understanding of intentionality are present in great apes. As such, right-handed gestures could be considered a more efficient means of information transfer than left-handed gestures.

This capacity to influence recipients in a goal-directed way through the use of right-handed gestures can increase the efficiency of social coordination. The fact that use of right-handed gestures was associated with a longer duration of unidirectional grooming relative to left-handed gestures suggests that the use of these gestures is important in goal-directed contexts, in which the signaler indicates the body parts the recipient should move. By indicating these body parts more precisely, the signalers can spatially disambiguate a referent among a set of potential targets and coordinate grooming more effectively. Further, the use of right-handed gestures in the contexts of higher mating success suggests that the ability to convey the goal of interaction accurately may rely on the ability to increase the accuracy of manual indication, in order to direct the movement and attention of the recipient more effectively. Chimpanzees that had more social partners with whom they maintained coordinated activities for longer durations (e.g., joint travel, mating), receive a higher rate of right-handed gestures. By directing right-handed gestures at the desired social partners, signalers may be able to more precisely indicate the social partner with whom they desire to coordinate behavior and direct their movement and attention towards the goal. This ability to more accurately indicate the target of interaction through right-handed gestures is evident in human communication. In humans, deficits in apraxia left-hemisphere-damaged patients result in deficits in target-aiming through right-handed reaching movements (Mutha et al. 2010). In accordance with this function of right-handed gestures in humans, the results of our study point at the role of the left hemisphere in controlling the execution of chimpanzee right-handed gestures.

Right-handed gestures appear to be more effective in coordinating social behavior relative to left-handed gestures when social bonds are weaker. When only dyads who did not mutually groom were considered, right-handed gestures were more effective in eliciting a response from the recipient. Furthermore, right-handed gestures were more effective than left-handed gestures in eliciting grooming reciprocity when chimpanzees did not engage in mutual grooming. The weaker social ties arising in multilevel societies are more challenging to manage, and in interactions with individuals who are weakly bonded, right-handed gestures may increase the efficiency of coordination. The fact that right-handed gestures were restricted in use to evolutionarily “urgent” contexts when the failure to accurately convey information may have important negative consequences, such as interactions with central individuals in the network, and in contexts of aggression, grooming, or mating, suggests that efficient communication through right-handed gestures may be a key factor driving fitness. The greater mating success of chimpanzees using these gestures, for instance, demonstrates the greater success of signalers using right-handed gestures to initiate mating. These data are the first to demonstrate that the fitness benefits of efficient information transfer when the social bonds are weaker may drive left-hemisphere bias for cortically controlled, right-handed gestures in evolutionarily “urgent” contexts (Fitch and Braccini 2013).

However, social coordination through right-handed gestures may have been challenging for chimpanzees when parties were larger, possibly because of the distraction imposed by the importance of monitoring information about third-party audience in these parties. The elaborations of right-handed gestures were more likely in smaller parties, when audiences of competitive partners were absent, suggesting that these interactions were cognitively challenging and prone to distraction. The cognitive complexity of establishing joint attention through right-handed gestures may have restricted the use of these gestures in more complex social settings (Adamson 1995). This is particularly the case for visual gestures that require greater attention to monitoring the communication channel, thus demanding that chimpanzees switch to communication that can bring goal of interaction into joint focus of attention without the need for mutual visual monitoring such as right handed tactile and auditory gestures (Roberts 2018; Roberts and Roberts 2016a, 2016b). In agreement with previously proposed lateralization of manual gestures in the chimpanzee brain, the use of right-handed gestures in coordination contexts suggests that chimpanzees have a left-hemisphere bias for complex, intentional gestural communication in simpler social settings (Fitch and Braccini 2013).

In large parties, social coordination through right-handed gestures may be constrained because of the difficulty of engaging in joint attention in goal-directed contexts. This is particularly the case when interacting in larger parties with non-kin or different-age partners; in these circumstances, chimpanzees are less likely to engage in interaction (Roberts 2018). Left-handed gestures appear to play a role in coordinating social interactions in more complex social settings such as larger parties (Connor 1992; Noë and Hammerstein 1994). Left-handed gestures are controlled through the right hemisphere and therefore may be better suited to expressing emotions than right-handed gestures, as is the case in humans (Sackeim et al. 1978). Further, left-handed gestures, but not right-handed gestures, are associated with increased complexity of gestural communication, such as the combined gestures consisting of two or more gestures produced simultaneously. Through increased complexity, left-handed gestures may play an important role in amplifying and accelerating processing of emotional states to facilitate emotional convergence and performance of behaviors important in social cohesion (Mendl et al. 2010; Owren and Rendall 2001).

In chimpanzees, the level of arousal experienced by the individual is expressed by the rate of self-scratching. The recipients of left-handed gestures have a reduced rate of scratching, suggesting that the recipients may have experienced reduced arousal when receiving these gestures. Left-handed gestures may have greater potential to coordinate social activities in large parties by being more rewarding for the recipient than right- handed gestures. The rewarding property of left-handed gestures may act to redirect the focus of attention of the recipient from the wider audience onto the signaler, enabling the pair to engage in longer bouts of social interaction.

In particular, tactile, left-handed gestures may facilitate social coordination in the presence of a large social audience, which could be mediated by an upsurge in endorphins, and which may reduce the recipient’s arousal. However, the use of left-handed, tactile gestures on a larger scale is constrained by the dyadic nature of this behavior. In contrast, synchronized calls accompanying loud auditory gestures may complement these behaviors to increase the complexity of social relationships. Our results show that chimpanzees used left-handed gestures accompanied by synchronized pant-hoots when an audience of social competitors was present. When these behaviors were included in the model, the duration of mutual grooming in large parties and in the presence of social competition was longer even though the duration of unidirectional grooming was shorter. Further, the association between these behaviors and reduced recipients’ scratching suggests that synchronized pant-hoot calls may increase social cohesion by reducing the recipient’s anxiety (Roberts and Roberts 2016b). Whereas in smaller parties chimpanzees develop social bonds through grooming reciprocity by use of right-handed gestures, the impediment in the establishment of joint attention during grooming in larger social parties, or when a competitive audience is present, may reduce the likelihood of grooming reciprocity, and these social bonding mechanisms function to facilitate social cohesion. Thus, contexts of social competition may have specifically precipitated the evolution of these different social bonding mechanisms. It could be argued that in our study use of left-handed gestures was inflexible and prone to the influence of audience presence on signaler’s arousal. Against this possibility, our data show that chimpanzees did not respond to the presence of an audience of same-age partners by increasing their use of left-handed gestures. Thus, the response was not driven by increased arousal in response to the presence of partners from the social group that were more attentive to the signaler. Instead, our data show that chimpanzees flexibly tailored their use of rewarding gestures in response to the presence of the audience with whom recipients had greater social interest and therefore were likely to choose them as target of social interaction over the signaler.

Complexity of communication may influence the size of the party that can be maintained. In large social parties, mutual attention is prone to distraction because of the presence of a wider audience, and this may limit the capacity of chimpanzees to service social relationships through left handed, visual gestures. Our study shows that right-handed gestures may help to break the ceiling imposed on social interactions through left-handed visual gestures. Figure 1b shows that left-handed visual gestures enable chimpanzees to maintain parties of approximately 5 individuals, whereas right-handed visual gestures increase this threshold to approximately 8 individuals. However, when chimpanzees have to overcome this size limit—for instance, when females come into estrous—one way to adjust to the changing complexity of their social environments is through the use of gestures that exploit similar reward mechanisms to those exploited through grooming. Our data show that tactile and auditory gestures may enable chimpanzees to increase party size to the upper limit of 13, enabling chimpanzees to break through the constraint imposed on complexity of social interactions through visual gestures. This is much larger than the average party size of just 5 individuals recorded for East African chimpanzees, suggesting that tactile and auditory gestures play an important role in facilitating social complexity.

Primate encephalization has been linked to sophisticated social cognition, which includes the maintenance of stable social ties across multiple behavioral contexts (Aiello and Dunbar 1993; Dunbar 1995). These skills are thought to promote cooperative social interactions and enable differentiated relationships to function in cohesive units. These results imply that one important aspect of these skills may be the capacity for servicing social relationships through right-handed gestures. This cognitive capacity is seen in the link between social coordination and the capacity to flexibly influence recipients through the use of intentional, right-handed gestures in simpler social settings. On the other hand, emotional expression has information value (Lindell 2013). Whereas emotional expression is often viewed as inflexible and midbrain-controlled, previous findings in both humans and other primates suggest that in affiliation contexts, emotional communication is cortically controlled (Lindell 2013; Packheiser et al. 2019). Here, specifically, we indicate that cortical control over emotional, left-handed gestures may underlie the cognitive complexity underpinning social relationships with unrelated individuals in complex social settings. These complex cognitive skills enable chimpanzees to maintain more complex social relationships by modifying the efficiency with which they can influence the behavior of the recipient in different social settings (Roberts and Roberts 2015). More broadly, this indicates that, as group size increased during human evolution, there may have been an increase in intentional gestures followed by increasing reliance on coordination of social interactions through communication that incorporates rewarding property in the signal. Thus, an increased ability to voluntarily capture a recipient’s interest through intentional and rewarding communication appears to facilitate life in larger and more complex social groups. The understanding that others have goals and intentions different from one’s own appears to be at the heart of this transition, as shown by the flexible adjustment of right- and left-handed gestures in parties of increasing size in relation to the implied risk of defection by the recipient. The evolution of complex understanding of intentionality in contexts of social competition may thus underpin the coevolution of group size and brain size in humans.

Our study adds to previous research by showing that laterality is flexible according to social context. These findings support a growing number of studies showing that laterality is subject to learning biases in both humans and primates (Karim et al. 2017; Schaafsma et al. 2008) and that laterality can vary according to a number of different factors such as gesture type and positional factors (Bourjade et al. 2013; Chapelain et al. 2012; Meunier et al. 2012). More broadly, our findings imply that the contextual focus of the study and categorization of non-verbal behavior as a communicative gesture will affect the probability to find a laterality bias. For instance, in humans and other primates, right-hand preference is stronger for communicative gestures than for non-communicative actions such as grasping objects (Meguerditchian and Vauclair 2006, 2009; Meunier et al. 2012). Similarly, the differences between captive and wild settings may influence the likelihood of finding laterality bias in the populations (Llorente et al. 2011; Lonsdorf and Hopkins 2005; Marchant and McGrew 1996; McGrew and Marchant 1997a, 1997b). Whereas some captive colonies of primates such as chimpanzees display right-handed bias (Hopkins et al. 2012), there appears to be a left-handed population bias or ambilaterality in the wild (Hobaiter and Byrne 2013; McGrew and Marchant 2001). Our results support the findings of these studies and suggest that the nature of differentiated social relationships in the wild influences laterality. The prevalence of right-handed gestures in captivity may reflect the higher rates of grooming between individuals who have a more urgent need to resolve conflict due to close proximity. In contrast, the lower spatial cohesion among wild chimpanzees facilitates differentiated social relationships and therefore more ambilateral gesturing in the wild.

The lateralization of mechanisms responsible for language evolution is the subject of debate; both hemispheres have motor control over manual gestures (Mutha et al. 2012). Left-hemisphere specialization for the ability to learn and coordinate motor actions effectively underpins both praxis and language production (speech/sign), and has an enhanced role in flexible adjustment, enabling more effective comprehension and learning of new sequences and skills, including language. On the other hand, the right hemisphere facilitates sensorimotor stabilization of ongoing actions and reflexes. This includes stopping at a goal position, suggesting that right-hemisphere circuits might play a role in the withdrawal of communication acts, another key ability underpinning language. Left-handed humans also have a left-hemisphere dominance for language processing (Knecht et al. 2000), and nonhuman primates that show population-level right-handedness for communicative gestures nonetheless do not possess language.

The laterality of the control mechanisms from which language evolved is therefore an unresolved question, and current theoretical accounts focus on motor-control hypotheses, including enhanced tool use, driving language evolution (Bradshaw and Rogers 1992), and social or communicative hypotheses suggest that enhanced social bonding on a larger scale drives language evolution (Aiello and Dunbar 1993). Our data add to these theoretical debates by suggesting that the driving force behind language evolution may be increasing complexity of social relationships by increasing the efficiency of social coordination. Our data appear to suggest that language evolution was preceded by increasing precision of manual indication, which facilitated more complex social interactions. In humans, discrete forms of gestures such as pointing can accurately refer to events or objects in the external environment. In chimpanzees, the importance of such skills has previously been shown in foraging tasks whereby use of indicative gestures such as pointing was more efficient in directing recipients to a food source than other types of gestures (Gonseth et al. 2017; Roberts et al. 2014b). In both humans (Butterworth 2003) and other primates (Krause 1997; Krause and Fouts 1997), indexical pointing with arm and index finger is believed to have emerged in response to a need for increasing precision in behavior. Thus, the efficiency of information transfer to facilitate social coordination appears to drive chimpanzee left-hemisphere bias for right-handed gestures (Fitch and Braccini 2013). These skills appear to be a relatively recent adaptation, since use of intentional, right-handed gestures may be limited to Hominoidea (Pollick and de Waal 2007). Thus, it is possible that the capacity to coordinate movement and attention through increased precision of manual indication has been a key characteristic of the complex cognitive and communication skills that led to language evolution.

In particular, the context of coordination of joint activity may have provided an impetus for the evolution of language from right-handed gestures. Language relies on learnt, ritualized signals, and previous studies showed that chimpanzees have the capacity to increase homogeneity in their repertoire of visual and tactile gestural communication in repeated one-to-one interactions (Roberts and Roberts 2017). By enabling more precise indication of the target location, right-handed gestures can indicate objects or events in the external environment more accurately. Such capacity could lead to a more effective indication of referents in the external environment by right-handed gestures, resulting in the establishment of joint reference between interactants about external objects or events. By making the association between gesture form and the referent more precise, recipients can attribute meanings to gesture forms and learn them more effectively. The fact that some right-handed gestures overlapped with the recipient's repertoire and some did not suggests that the route to gesture innovation and learning is through right-handed gestures. 

By directing gestures at the recipient that are not in the recipient’s repertoire, signalers can make the target (e.g., a specific place on the body of the recipient) stand out among other potential targets, therefore making the signaler’s goal more apparent. Use of right-handed gestures is correlated with the use of low-amplitude calls such as panting and nonvocal sounds such as lip-smacks. These sounds are flexible and intentional, displaying direct cortical connection, but they are also homogenous and context-specific. Combined with these sounds, pointing out areas of the body through right-handed gestures may have provided the arena for the evolution of language. For instance, the acquisition of language in children is preceded by the ability to simultaneously attend to another person and to an object in the external environment. When the signaler (a parent or caregiver) points to and names the object in the external environment, the recipient (infant) can associate the object with its name, leading to the development of language (Butterworth 2003). In the case of chimpanzee gesturing, right-handed gestures can more precisely connect a spot on the body to the concurrent sounds to make a connection between the two. Thus, right-handed gestures not only indicate the spot on the body but also reinforce the link between spot on the body and the sound. Leavens and colleagues have argued that for such links to be formed, such acts would reliably receive positive emotional responses and thus grooming would provide an ideal context for making these connections (Leavens 2004; Leavens and Racine 2009; Leavens et al. 2005, 2009). Right-handed gestures allow the spot on the body to be identified with an auditory signal, which may play an important role in the evolution of language. The fact that lip-smacks have a coordination function in nature (Fedurek et al. 2015) suggests that the combined use of right-handed gestures with lip-smacks may enhance responsiveness to right-handed gestures, by further specifying the goal of the interaction to the recipient. For instance, studies have shown that the action of social neuro-hormones can automatically upregulate the mental capacity of the recipient to infer meaning from the behavior (Domes et al. 2007). In addition, the rewarding property of lip-smacks may increase commitment to the interaction, increasing the threshold of size of social parties that can be maintained.

In summary, our results strongly demonstrate that laterality is context-dependent, suggesting that divergence in manual lateralization between other primates and humans is affected by external factors such as the complexity of the social environment. Whereas left-handed gestures have a rewarding value in complex social settings, right-handed gestures increase comprehension of the gesture in simpler social settings. The flexible adjustment of manual laterality in parties of increasing size appears to be accompanied by a complex understanding that recipients have goals and intentions different from signalers, contingent upon the presence of a wider audience. If language evolved as a result of the selection pressures arising from complex sociality, then right-handed gestures may have played an important role by enabling more efficient coordination through increasing the comprehension of communication. These results show the potential social benefits of laterality and support the hypothesis that language evolved from right-handed gestures, accompanied by multimodal communication, to increase the efficiency of social coordination. Cross-cultural and cross-species comparisons will reveal the importance of the laterality of hand signals in language evolution.

## Electronic supplementary material

ESM 1 (DOCX 38 kb)

ESM 2 (DOCX 83 kb)

ESM 3 (DOCX 14 kb)

ESM 4 (DOCX 13 kb)

ESM 5 (DOCX 17 kb)

ESM 6 (DOCX 13 kb)
