# Supplementary material for: Complex Sociality of Wild Chimpanzees Can Emerge from Laterality of Manual Gestures
Source: Hum Nat. 2019 Jun 24;30(3):299–325. doi: 10.1007/s12110-019-09347-3 (PMC6698263; doi:10.1007/s12110-019-09347-3)
Supplement: Supplementary file 3 — (DOCX 38 kb) [file 12110_2019_9347_MOESM1_ESM.docx]

Electronic Supplementary Material (ESM) - 1

for

Complex Sociality of Wild Chimpanzees Can Emerge from Laterality of Manual Gestures

Anna Ilona Roberts, Lindsay Murray, Sam George Bradley Roberts

*Human Nature* 30(3), 2019. Doi: to be added in proofs.

Table S1. Identity of the focal subject, their sex, approximate age, reproductive status of the 12 focal subjects included in the study.

| Focal subject ID | Sex | Age | Female reproductive status | Total observation duration (hours) |
| --- | --- | --- | --- | --- |
| BB | Male | 21 |  | 8.60 |
| HW | Male | 15 |  | 17.17 |
| KT | Male | 15 |  | 17.10 |
| KU | Female | 29 | Pregnant | 15.17 |
| KW | Female | 27 | Nursing | 8.50 |
| ML | Female | 33 | Cycling | 18.63 |
| MS | Male | 17 |  | 8.73 |
| NB^b^ | Female | 46 | Cycling | 8.33 |
| NK^a^ | Male | 26 |  | 9.70 |
| RH | Female | 43 | Nursing | 17.30 |
| SQ | Male | 17 |  | 9.23 |
| ZM | Female | 40 | Cycling | 11.83 |

Notes. ^a^ Alpha male^, b^ Alpha female

Table S2. Definitions and descriptive data for variables entered into Generalized Linear Mixed Models, based on 12 chimpanzees. All social behaviours measured as durations (mins), per hour dyad spent in same party. For joint behaviours (feeding, resting, travelling) both dyad partners were engaged in the same behaviour.

| Behaviour | Definition | Mean ± s.d. or  presence/absence |
| --- | --- | --- |
| Sex difference | Sex difference between focal subject and the recipient (0 = different sex, 1 = same sex) | 0 = 84, 1 = 64 |
| Age difference | Age difference between focal subject and the recipient (0 = different age, 1 = same age) | 0 = 126, 1 = 22 |
| Reproductive state difference | Reproductive state difference between focal subject and the recipient (0 = different reproductive state: unoestrous female-oestrous female, unoestrous female-male dyad; 1 = same reproductive state: male-male, male-oestrous female, oestrous female – male, unoestrous female – unoestrous female, oestrous female- oestrous female dyad) | 0 = 27, 1 = 107 |
| Maternal kinship | Maternal kinship presence between focal subject and the recipient (0 = absent, 1 = present) | 0 = 142, 1 = 6 |
| Audience same age as focal | Presence of same age partners in the audience within 10 meters as the signaller (0 = absent, 1 = present) during production of the gesture | 0 = 74, 1 = 56 |
| Audience same age as recipient | Presence of same age partners in the audience within 10 meters as the recipient (0 = absent, 1 = present) during production of the gesture | 0 = 93, 1 = 37 |
| Party size | Total number of adult, subadult, juvenile and infant individuals present in the party during production of the gesture | 8.76 ±6.12 |
| Joint feeding | Duration of jointly feeding when nearest neighbours and within 2 m | 0.99 ±1.61 |
| Joint resting | Duration of jointly resting when nearest neighbours and within 2 m | 1.70 ±2.83 |
| Joint travel | Duration of jointly travelling when nearest neighbours and within 2 m | 0.48 ±1.14 |
| Unidirectional grooming | Duration of unidirectional grooming to the dyad partner | 1.42 ± 2.30 |
| Grooming received | Duration of grooming received from the dyad partner | 1.08 ± 2.33 |
| Grooming mutual | Duration of mutually grooming with the dyad partner | 1.26 ± 2.64 |
| Laterality | Manual gesture made with a left hand (left-handed, 0) or right hand (right-handed, 1) | 0 = 72  1 = 76 |
| Left-handed repertoire size | Repertoire size of left-handed gestures within the sequences where manual gestures were present | 0.52 ± 0.59 |
| Right-handed repertoire size | Repertoire size of right-handed gestures within the sequences where manual gestures were present | 0.54 ± 0.55 |
| Response by activity change or communication | Change of recipients behaviour following the gesture or communication (e.g. display produced by the recipient after the gesture which is not followed by change in recipients behaviour such as embrace during travel, whereby signallers travel immediately before and after the embrace). If both response by activity change and communication co-occurred (e.g. vocalise and starts to groom) this was categorized as response by activity change: 0 = activity, 1 = communication | 0 = 60, 1 = 29 |
| Response present or absent | Presence (1) or absence (0) of a change in recipients behaviour following the gesture | 0 = 208, 1 = 258 |
| Right-handed gesture frequency | Number of right-handed gestures within a sequence | 0.18 ± 0.52 |
| Left-handed gesture frequency | Number of left-handed gestures within a sequence | 0.16 ± 0.42 |
| Right-handed visual gesture frequency | Number of visual right-handed gestures within a sequence where manual gestures are present | 0.18 ± 0.38 |
| Right-handed tactile gesture frequency | Number of tactile right-handed gestures within a sequence where manual gestures are present | 0.13 ± 0.36 |
| Right-handed auditory short-range gesture frequency | Number of auditory short-range right-handed gestures within a sequence where manual gestures are present | 0.07 ± 0.53 |
| Right-handed auditory long-range gesture frequency | Number of auditory long-range right-handed gestures within a sequence where manual gestures are present | 0.25 ± 0.54 |
| Left-handed visual gesture frequency | Number of visual left-handed gestures within a sequence where manual gestures are present | 0.18 ± 0.42 |
| Left -handed tactile gesture frequency | Number of tactile left-handed gestures within a sequence where manual gestures are present | 0.33 ± 0.47 |
| Left -handed auditory short-range gesture frequency | Number of auditory short-range left-handed gestures within a sequence where manual gestures are present | 0.006 ± 0.08 |
| Left -handed auditory long-range gesture frequency | Number of auditory long-range left-handed gestures within a sequence where manual gestures are present | 0.15 ± 0.37 |
| Communicative repair and other communication | Communicative repair by elaboration whereby signaller produces another gesture type when the initial gesture has been unsuccessful in eliciting desired response (1) versus all other communication types combined (0) | 0 = 139, 1 = 11 |
| Communicative repair and other sequence | Communicative repair by elaboration whereby signaller produces another gesture type when the initial gesture has been unsuccessful in eliciting desired response (1) versus all other sequence types combined (0) | 0 = 51, 1 = 11 |
| Reciprocity | Bout of giving grooming is either 0 - non-reciprocated (recipient of unidirectional grooming does not reciprocate by grooming unidirectionally or mutually grooming), 1 – reciprocated (recipient of grooming grooms back the groomee unidirectionally or by engaging in mutual grooming) | 0 = 51, 1 = 17 |
| Dyad type | Adult focal subject gives grooming to: 0 – individual with whom they were not observed to mutually groom during study period, 1 – individual with whom they mutually groom | 0 = 19, 1 = 43 |
| Laterality grooming | Presence of left-handed (0) or right-handed (1) gestures within a grooming bout prior to reciprocity or cessation of grooming | 0 = 2, 1 = 12 |
| Visual gesture | Number of gestures in the sequence whereby perception of gesture is only possible by looking at the signaller (exludes cases when panthoot was recorded) | 0.94 ± 1.52 |
| Auditory short-range gesture | Number of gestures in the sequence whereby sounds produced by the gesture can be heard within 10 m of the signaller (excludes lipsmack) | 0.04 ± 0.33 |
| Auditory long-range gesture | Number of gestures in the sequence whereby sounds produced by the gesture can be heard over 10 m from the signaller (exludes cases when panthoot was recorded) | 0.36 ± 1.60 |
| Synchronized high-intensity panthoot | Pant-hoot call produced jointly with other group members and accompanied by simultaneous production of auditory gestures, which produce sounds audible at a distance of at least 10 meters independently of the acoustic properties of the pant-hoot call. If both visual and auditory gestures simultaneously accompanied the pant-hoot call within the same sequence it was scored as high-intensity. 0 = absent, 1 = present | 0 = 524, 1 = 17 |
| Synchronized low-intensity panthoot | Pant-hoot call produced jointly with other group members and accompanied by simultaneous production of visual gestures, which can be perceived only by looking at signaller. 0 = absent, 1 = present | 0 = 537, 1 = 8 |
| Lip smack | Sounds made using lips, oral cavity or teeth, without the use of the vocal tract such as splutters and teeth chomps. | 0.28 ± 0.57 |

Table S3. Definitions, means and standard deviations ± s.d. or presence absence of events entered into MRQAP regressions. Data based on social behaviour and gestural communication between 132 chimpanzee dyads. All gestural communication measured as the rate per hour dyad spent within 10 m. All social behaviors measured as the rate per hour spent in the same party.

| Gesture | | Definition | | Mean ± s.d. or  presence/absence | |
| --- | --- | --- | --- | --- | --- |
| Sex difference | | Sex difference between focal subject and the recipient (0 = different sex, 1 = same sex) | | 0 = 60  1 = 72 | |
| Age difference | | Age difference between focal subject and the recipient (0 = different age, 1 = same age) | | 0 = 102  1 = 30 | |
| Reproductive state difference | | Reproductive state difference between focal subject and the recipient (0 = different reproductive state: unoestrous female-oestrous female, unoestrous female-male dyad; 1 = same reproductive state: male-male, male-oestrous female, oestrous female – male, unoestrous female – unoestrous female, oestrous female- oestrous female dyad) | | 0 = 54  1 = 78 | |
| Maternal kinship | | Maternal kinship presence between focal subject and the recipient (0 = absent, 1 = present) | | 0 = 126  1 = 6 | |
| Joint feeding | | Duration of jointly feeding when nearest neighbours and within 2 m | | 0.62±1.36 | |
| Joint resting | | Duration of jointly resting when nearest neighbours and within 2 m | | 0.99±5.30 | |
| Joint travel | | Duration of jointly travelling when nearest neighbours and within 2 m | | 0.24±0.91 | |
| Unidirectional grooming | | Duration of unidirectional grooming to the dyad partner | | 0.34±1.09 | |
| Grooming received | | Duration of grooming received from the dyad partner | | 0.32±1.39 | |
| Grooming mutual | | Duration of mutually grooming with the dyad partner | | 0.37±1.63 | |
| Attention present | | Duration of mutual bodily orientation presence when nearest neighbours and within 2 m of the dyad partner | | 1.57 ± 3.66 | |
| Attention absent | | Duration of mutual bodily orientation absence when nearest neighbours and within 2 meters | | 1.84 ± 5.68 | |
| Proximity to 2 meters | | Duration of proximity when nearest neighbours and within 2 m of the dyad partner | | 3.42 ± 7.43 | |
| Proximity to 10 meters | | Duration of proximity within 10 m of the dyad partner | | 23.09 ± 14.21 | |
| Mating | | Copulation of the male with the female | | 0.14 ± 0.79 | |
| Scratch produced | | Individual rakes with the fingers through the fur and skin repeatedly or singly – all individuals within 10 meters were coded as recipients of this behaviour. | | 0.64 ± 1.68 | |
| Scratch received | | Focal subject receives the scratch whereby the non-focal subject rakes with the fingers through the fur and skin repeatedly or singly | | 0.64 ± 1.68 | |
| Right-handed | | Manual gesture made with a right hand | | 0.18 ± 0.60 | |
| Left-handed | | Manual gesture made with a left hand | | 0.41 ± 2.16 | |
| Bodily | | A gesture is produced by the signaller with the part of the body (e.g. head, legs, torso) that does not involve use of hands | | 2.52 ± 6.80 | |
| Manual | | A gesture is made exclusively with the hand | | 0.93 ± 3.19 | |
| Non-combined gesture | | A single gesture is produced by the signaller without other accompanying gesture type | | 2.47 ± 6.60 | |
| Combined gesture | | Two or more gestures are produced simultaneously by the signaller (e.g. embrace and thrust) | | 0.46 ± 1.70 | |
| Gesture with no object | | Gesture is produced not using object | | 2.56 ± 7.14 | |
| Gesture with object | | Gesture is produced using object (e.g. shake branch) | | 0.88 ± 3.12 | |
| Events | | Number of consecutive gesture events in the sequence. One gesture event can contain gestures combined or not combined with other gestures (e.g. embrace and thrust co-occurring would be counted as one event) | | 3.18 ± 7.82 | |
| Dyadic repertoire size | | The number of gesture types produced towards the dyad partner, per hour spent within 10 m | | 1.97 ± 5.11 | |
| Indicative | | Movement of the arm and hand towards the recipient, without physical touch or contact with substrate | | 0.14 ± 0.54 | |
| Non-indicative | | Movement of the arm and hand that involves physical touch or contact with the substrate or visual but does not involve movement of the hand towards the recipient | | 0.80 ± 3.01 | |
| Unimodal gesture | | Gesture does not include accompanying facial expression or vocalization | | 1.79 ± 5.81 | |
| Multimodal gesture (facial expression) | | Gesture accompanied by simultaneous production of facial expression | | 0.09 ± 0.52 | |
| Mulitmodal gesture (low amplitude vocalization) | | A lower amplitude vocalization other than panthoot is produced whilst the signaller is gesturing | | 0.25 ± 0.87 | |
| Mulitmodal gesture (high amplitude vocalization) | | A high amplitude vocalization such as panthoot call is produced whilst the signaller is gesturing | | 0.81 ± 3.25 | |
| Gesture with mutual attention absent | | Gesture is not accompanied by simultaneous presence of mutual visual contact between signaller and the recipient. Mutual visual contact is when signaller’s and recipient’s body are within each other’s field of view (up to 45 degrees body turn) | | 0.78 ± 3.39 | |
| Gesture with mutual attention present | | Gesture is accompanied by simultaneous presence of mutual visual contact between signaller and the recipient. | | 1.23 ± 4.15 | |
| Penile erection | | Production of a gesture is accompanied by simultaneous erection of the penis by the signaller | | 0.19 ± 0.99 | |
| Piloerection | | Production of a gesture is accompanied by simultaneous involuntary erection of hairs | | 1.21 ± 5.11 | |
| Non repetitive gesture | | A gesture that does not involve repetition of movement in regular and cyclical fashion such as static presentation of a torso for grooming | | 1.82 ± 5.72 | |
| Repetitive gesture | | A gesture involves repetition of movement in regular and cyclical fashion in predictable manner that indicates that the movement forms part of one gesture | | 1.69 ± 4.80 | |
| Homogenous gesture | | Gesture type is present in both signaller’s and recipient’s repertoire of gestures | | 1.76 ± 5.49 | |
| Heterogeneous gesture | | Gesture type occurs only in signaller’s repertoire of gestures | | 0.72 ± 2.64 | |
| Single gesture | | Signaller produces one gesture (accompanied or not by another) towards the same recipient, with the same goal, within the same context, and made within a maximum of 30 seconds interval to ensure independence. | | 1.27 ± 4.07 | |
| Rapid sequence | | When a chimpanzee produces more than one gesture consecutively within a sequence and there is no intermittent pause between gestures, then the chimpanzee has produced a ‘rapid sequence’. | | 0.45 ± 1.30 | |
| Persistence sequence | | Persistence of gesturing is when the chimpanzee produces one gesture or a gesture sequence, then after a period of response waiting (1-5s) they produce another gesture - here such instances are termed a ‘persistence sequence’. | | 0.11 ± 0.45 | |
| Repetition | | Sequence of gestures within persistence sequence is composed of one gesture type | | 0.03 ± 0.24 | |
| Elaboration | | Sequence of gestures within persistence sequence is composed of more than one gesture type | | 0.16 ± 0.53 | |
| Close proximity | | Signaler produced a gesture within 1 meter from the recipient | | 1.09 ± 4.40 | |
| Far proximity | | Signaler produced a gesture from above 1 meter away from the recipient | | 1.64 ± 5.03 | |
| Visual gesture | | Perception of gesture is only possible by looking at the signaller | | 1.97 ± 5.54 | |
| Auditory short-range gesture | | Sounds produced by the gesture can be heard within 10 m of the signaller | | 0.41 ± 2.33 | |
| Auditory long-range gesture | | Sounds produced by the gesture can be heard over 10 m from the signaller | | 0.67 ± 2.30 | |
| Tactile gesture | | Perception of the gesture is possible via physical contact | | 0.44 ± 2.43 | |
| Response present | | Presence of any change in the behaviour of the recipient following production of the gesture | | 0.94 ± 3.64 | |
| Response absent | | Absence of any change in the behaviour of the recipient following production of the gesture | | 0.58 ± 2.36 | |
| Lip smack | | Sounds made using lips, oral cavity or teeth, without the use of the vocal tract such as splutters and teeth chomps. | | 0.35 ± 1.92 | |
| Threat to dominate | | Aggressive context, where there is no tangible reason for conflict but the recipient reacts with fear (e.g. screams) | | 0.07 ± 0.66 |  |
| Other threat | | Communication motivated by clear conflict of interest over the resource such as food or behavior such as mating | | 0.07 ± 0.36 |  |
| Copulation | | Gestures produced by a male or a tumescent female in order to initiate copulation | | 0.14 ± 0.79 |  |
| Greeting | | Gestures accompanying approaching, being approached or leaving approach with the recipient who is non-threatening or when the recipient or third party distressed, frightened or hurt the signaller. | | 0.27 ± 0.74 |  |
| Mutual groom | | Gestures made to initiate simultaneous grooming between signaller and the recipient. | | 0.07 ± 0.66 |  |
| Receive groom | | Gestures made to initiate grooming of the signaller by the recipient. | | 0.19 ± 0.80 |  |
| Give groom | | Gestures made to initiate grooming of the recipient by the signaller. | | 0.37 ±1.94 |  |
| Synchronized low-intensity panthoot | | Pant-hoot call produced jointly with other group members and accompanied by simultaneous production of visual gestures, which can be perceived only by looking at signaller. | | 0.05 ± 0.36 |  |
| Solo high-intensity panthoot | | Pant-hoot call produced solo (without joining in by other group members) and accompanied by simultaneous production of auditory gestures, which produce sounds audible at a distance of at least 10 meters independently of the acoustic properties of the pant-hoot call. If both visual and auditory gestures simultaneously accompanied the pant-hoot call within the same sequence it was scored as high-intensity. | | 0.08 ± 0.47 |  |
| Synchronized high-intensity panthoot | | Pant-hoot call produced jointly with other group members and accompanied by simultaneous production of auditory gestures, which produce sounds audible at a distance of at least 10 meters independently of the acoustic properties of the pant-hoot call. If both visual and auditory gestures simultaneously accompanied the pant-hoot call within the same sequence it was scored as high-intensity. | | 0.20 ±1.0 |  |

Table S4. Relationship between total observation time of the focal subject attribute and dyadic laterality networks. Geary’s C statistic values smaller than 1 indicate positive correlation, a value of 1 indicates perfect independence, larger values than 1 indicate negative correlation.

| Gesture network | Geary’s statistic | P value |
| --- | --- | --- |
| Left-handed | 1.319 | 0.269 |
| Right-handed | 0.896 | 0.388 |
