# Supplementary material for: Complex Sociality of Wild Chimpanzees Can Emerge from Laterality of Manual Gestures
Source: Hum Nat. 2019 Jun 24;30(3):299–325. doi: 10.1007/s12110-019-09347-3 (PMC6698263; doi:10.1007/s12110-019-09347-3)
Supplement: Supplementary file 4 — (DOCX 83 kb) [file 12110_2019_9347_MOESM2_ESM.docx]

Electronic Supplementary Material (ESM) - 2

for

Complex Sociality of Wild Chimpanzees Can Emerge from Laterality of Manual Gestures

Anna Ilona Roberts, Lindsay Murray, Sam George Bradley Roberts

*Human Nature* 30(3), 2019. Doi: to be added in proofs.

# Details of Results

Table S1. Results of GLMMs comparing response by activity change versus response by communication

| Independent variable | Standardized coefficient | Standard error | P value |
| --- | --- | --- | --- |
| Laterality | 0.647 | 0.295 | **0.031** |

Table S2. Results of GLMMs comparing response present and absent in relation to the frequency of right handed and left handed gestures within a sequence

| Independent variable | Standardized coefficient | Standard error | P value |
| --- | --- | --- | --- |
| Right-handed gesture frequency | 0.462 | 0.249 | 0.064 |
| Left-handed gesture frequency | 0.815 | 0.303 | **0.007** |

Table S3. Results of GLMMs comparing response present and absent between dyads who mutually groomed

| Independent variable | Standardized coefficient | Standard error | P value |
| --- | --- | --- | --- |
| Laterality | 1.284 | 0.711 | 0.077 |

Table S4. Results of GLMMs comparing response present and absent between dyads who did not mutually groom

| Independent variable | Standardized coefficient | Standard error | P value |
| --- | --- | --- | --- |
| Laterality | -0.710 | 0.318 | **0.029** |

Table S5. Results of GLMMs comparing presence of reciprocity to unidirectional grooming bout present and absent in relation to laterality of gesture used within the bout and presence or absence of mutual grooming within the dyad

| Independent variable | Standardized coefficient | Standard error | P value |
| --- | --- | --- | --- |
| Laterality | -22.759 | 0.840 | 0.001 |
| Dyad type | -22.606 | 0.941 | 0.001 |

Table S6. Results of GLMMs comparing right handed and left handed gestures in relation to reciprocity present and absent and dyad type

| Independent variable | Standardized coefficient | Standard error | P value |
| --- | --- | --- | --- |
| Dyad type | 22.788 | 1.072 | 0.001 |
| Reciprocity | -22.273 | 4.145 | 0.001 |

Table S7. Results of GLMMs comparing presence of communicative repair sequence versus all other communication types.

| Independent variable | Standardized coefficient | Standard error | P value |
| --- | --- | --- | --- |
| Sex difference | -0.037 | 0.647 | 0.955 |
| Reproductive status | -0.847 | 0.692 | 0.209 |
| Age difference | 0.873 | 1.012 | 0.390 |
| Maternal kinship | 10.886 | 0.443 | **<0.001** |
| Laterality | -1.325 | 0.446 | **0.004** |

Table S8. Results of GLMMs comparing presence of communicative repair sequence versus all sequence types.

| Independent variable | Standardized coefficient | Standard error | P value |
| --- | --- | --- | --- |
| Sex difference | 0.656 | 0.506 | 0.202 |
| Reproductive status | -1.098 | 0.619 | 0.083 |
| Age difference | 0.364 | 0.719 | 0.615 |
| Maternal kinship | 8.001 | 0.454 | **<0.001** |
| Laterality | -1.210 | 0.435 | **0.008** |

Table S9. Results of GLMMs comparing left versus right manual laterality.

| Independent variable | Standardized coefficient | Standard error | P value |
| --- | --- | --- | --- |
| Maternal kinship | -14.578 | 1.396 | **<0.001** |
| Sex difference | 0.188 | 0.364 | 0.607 |
| Age difference | -0.986 | 0.268 | **<0.001** |
| Reproductive status | -0.100 | 0.367 | 0.786 |
| Visual | -0.026 | 0.121 | 0.831 |
| Tactile | -0.783 | 0.241 | **0.002** |
| Auditory short-range | 0.388 | 0.245 | 0.116 |
| Auditory long-range | -0.042 | 0.086 | 0.624 |
| Lip smack | 4.005 | 0.266 | **<0.001** |
| Synchronized high intensity panthoot | 1.522 | 0.473 | **0.002** |
| Synchronized low intensity panthoot | 14.066 | 0.261 | **<0.001** |
| Unidirectional grooming | 0.199 | 0.144 | 0.170 |
| Grooming received | 0.134 | 0.059 | **0.025** |
| Grooming mutual | -0.317 | 0.136 | **0.021** |
| Joint feeding | -0.011 | 0.161 | 0.947 |
| Joint resting | 0.010 | 0.065 | 0.879 |
| Joint travel | -0.021 | 0.190 | 0.914 |
| Party size | -0.057 | 0.025 | **0.026** |

Table S10. Results of GLMMs comparing left versus right manual laterality.

| Independent variable | Standardized coefficient | Standard error | P value |
| --- | --- | --- | --- |
| Maternal kinship | -18.662 | 1.577 | **<0.001** |
| Sex difference | -0.004 | 0.502 | 0.994 |
| Age difference | -1.156 | 0.497 | **0.022** |
| Reproductive status | 0.219 | 0.529 | 0.680 |
| Visual | -0.025 | 0.109 | 0.821 |
| Tactile | -0.648 | 0.177 | **<0.001** |
| Auditory short-range | 0.356 | 0.254 | 0.163 |
| Auditory long-range | -0.058 | 0.075 | 0.443 |
| Lip smack | 5.084 | 0.201 | **<0.001** |
| Synchronized high intensity panthoot | 1.479 | 0.720 | **0.042** |
| Synchronized low intensity panthoot | 15.907 | 0.545 | **<0.001** |
| Unidirectional grooming | 0.187 | 0.170 | 0.276 |
| Grooming received | 0.224 | 0.074 | **0.003** |
| Grooming mutual | -0.343 | 0.167 | **0.043** |
| Joint feeding | -0.043 | 0.158 | 0.785 |
| Joint resting | 0.035 | 0.050 | 0.487 |
| Joint travel | -0.067 | 0.202 | 0.741 |
| Audience same age as recipient | 0.914 | 0.370 | **0.015** |
| Audience same age as focal | -0.284 | 0.477 | 0.553 |

Table S11. Results of GLMM examining factors influencing party size

| Independent variable | Standardized coefficient | Standard error | P value |
| --- | --- | --- | --- |
| Sex difference | 0.578 | 0.369 | 0.120 |
| Reproductive status | -2.201 | 2.088 | 0.294 |
| Maternal kinship | -10.200 | 3.118 | **0.001** |
| Age difference | -1.190 | 1.454 | 0.414 |
| Repertoire size of left-handed gestures | -1.473 | 1.769 | 0.407 |
| Repertoire size of right-handed gestures | -3.711 | 1.141 | **0.001** |

Table S12. Results of GLMM examining factors predicting presence and absence of audience of same age as the recipient

| Independent variable | Standardized coefficient | Standard error | P value |
| --- | --- | --- | --- |
| Sex difference | -1.358 | 0.953 | 0.157 |
| Reproductive status | -0.222 | 1.452 | 0.879 |
| Maternal kinship | -3.228 | 1.065 | **0.003** |
| Age difference | -0.217 | 1.185 | 0.855 |
| Repertoire size of left-handed gestures | -0.960 | 0.629 | 0.129 |
| Repertoire size of right-handed gestures | -1.605 | 0.568 | **0.006** |

Table S13. Results of GLMM examining factors predicting presence and absence of audience of same age as the focal subject

| Independent variable | Standardized coefficient | Standard error | P value |
| --- | --- | --- | --- |
| Sex difference | -0.376 | 0.519 | 0.471 |
| Reproductive status | -1.184 | 0.670 | 0.080 |
| Maternal kinship | -2.709 | 1.705 | 0.115 |
| Age difference | 0.188 | 0.625 | 0.764 |
| Repertoire size of left-handed gestures | -0.430 | 0.463 | 0.355 |
| Repertoire size of right-handed gestures | -0.828 | 0.324 | **0.012** |

Table S14. Results of GLMM examining laterality of gestures categorised according to modality influencing party size

| Independent variable | Standardized coefficient | Standard error | P value |
| --- | --- | --- | --- |
| Sex difference | 0.466 | 0.563 | 0.409 |
| Reproductive status | -1.806 | 2.081 | 0.387 |
| Age difference | -1.177 | 1.440 | 0.415 |
| Maternal kinship | -8.980 | 2.514 | **0.001** |
| Left-handed visual | -2.420 | 1.286 | 0.062 |
| Left-handed auditory short-range | 3.048 | 2.117 | 0.152 |
| Left-handed auditory long-range | 1.087 | 1.401 | 0.439 |
| Left-handed tactile | -0.982 | 1.081 | 0.365 |
| Right-handed visual | -2.507 | 0.578 | **<0.001** |
| Right-handed tactile | -0.995 | 1.571 | 0.528 |
| Right-handed auditory long-range | -4.117 | 0.887 | **<0.001** |
| Right-handed auditory short-range | -0.171 | 0.412 | 0.679 |

# Dyadic Social Behaviour and Laterality

Table S15. MRQAP regression models predicting durations of social behavior, per hour dyad spent in the same party or rate of social behaviour. Predictors were left-handed and right-handed gestures. Dyads were classified as same age or different age (within 5 years), same sex or different sex, related by maternal kinship and as the same or different reproductive status (reproductively active, not reproductively active). Based on 132 dyadic relationships of the chimpanzees. Significant *p* values are indicated in bold.

Table S15.1 Duration of attention away behaviour (*r^2^* = 0.089)

| Independent variable | Standardized coefficient | Standard error | *p* |
| --- | --- | --- | --- |
| Age | 0.312 | 1.461 | **0.003** |
| Sex | -0.114 | 1.146 | 0.102 |
| Kinship | 0.039 | 2.512 | 0.174 |
| Reproductive status | -0.034 | 1.137 | 0.340 |
| Left-handed gesture | -0.005 | 0.260 | 0.520 |
| Right-handed gesture | 0.099 | 1.032 | 0.089 |

Table S15.2 Duration of attention towards behaviour (*r^2^* = 0.167)

| Independent variable | Standardized coefficient | Standard error | *p* |
| --- | --- | --- | --- |
| Age | 0.160 | 1.002 | 0.076 |
| Sex | 0.054 | 0.774 | 0.288 |
| Kinship | 0.178 | 1.828 | 0.076 |
| Reproductive status | 0.223 | 0.967 | **0.047** |
| Left-handed gesture | 0.204 | 0.155 | **0.025** |
| Right-handed gesture | 0.026 | 0.588 | 0.287 |

Table S15.3 Duration of proximity to 2 meters (*r^2^* = 0.129)

| Independent variable | Standardized coefficient | Standard error | *p* |
| --- | --- | --- | --- |
| Age | 0.318 | 1.895 | **0.001** |
| Sex | -0.061 | 1.438 | 0.260 |
| Kinship | 0.118 | 3.259 | 0.100 |
| Reproductive status | 0.084 | 1.806 | 0.257 |
| Left-handed gesture | 0.097 | 0.344 | 0.091 |
| Right-handed gesture | 0.089 | 1.247 | 0.139 |

Table S15.4 Duration of joint feeding (*r^2^* = 0.137)

| Independent variable | Standardized coefficient | Standard error | *p* |
| --- | --- | --- | --- |
| Age | 0.026 | 0.326 | 0.379 |
| Sex | 0.088 | 0.295 | 0.207 |
| Kinship | 0.287 | 0.662 | **0.015** |
| Reproductive status | 0.225 | 0.288 | **0.004** |
| Left-handed gesture | -0.102 | 0.063 | 0.105 |
| Right-handed gesture | 0.071 | 0.235 | 0.189 |

Table S15.5 Duration of joint resting (*r^2^* = 0.069)

| Independent variable | Standardized coefficient | Standard error | *p* |
| --- | --- | --- | --- |
| Age | 0.276 | 1.295 | **0.002** |
| Sex | -0.160 | 1.067 | **0.022** |
| Kinship | -0.025 | 2.191 | 0.387 |
| Reproductive status | -0.072 | 1.032 | 0.288 |
| Left-handed gesture | -0.009 | 0.244 | 0.486 |
| Right-handed gesture | 0.060 | 0.948 | 0.103 |

Table S15.6 Duration of joint travel (*r^2^* = 0.121)

| Independent variable | Standardized coefficient | Standard error | *p* |
| --- | --- | --- | --- |
| Age | 0.261 | 0.253 | **0.017** |
| Sex | 0.025 | 0.209 | 0.388 |
| Kinship | 0.009 | 0.475 | 0.331 |
| Reproductive status | 0.017 | 0.203 | 0.463 |
| Left-handed gesture | 0.205 | 0.043 | **0.034** |
| Right-handed gesture | 0.011 | 0.146 | 0.338 |

Table S15.7 Duration of groom give (*r^2^* = 0.247)

| Independent variable | Standardized coefficient | Standard error | *p* |
| --- | --- | --- | --- |
| Age | 0.258 | 0.244 | **0.004** |
| Sex | 0.030 | 0.190 | 0.334 |
| Kinship | 0.152 | 0.444 | 0.066 |
| Reproductive status | 0.083 | 0.219 | 0.211 |
| Left-handed gesture | 0.153 | 0.051 | 0.067 |
| Right-handed gesture | 0.278 | 0.182 | **0.015** |

Table S15.8 Duration of groom mutual (*r^2^* = 0.114)

| Independent variable | Standardized coefficient | Standard error | *p* |
| --- | --- | --- | --- |
| Age | 0.215 | 0.462 | **0.047** |
| Sex | -0.049 | 0.393 | 0.354 |
| Kinship | 0.042 | 0.842 | 0.204 |
| Reproductive status | 0.117 | 0.410 | 0.175 |
| Left-handed gesture | 0.241 | 0.081 | **0.026** |
| Right-handed gesture | -0.003 | 0.265 | 0.608 |

Table S15.9 Duration of groom receive (*r^2^* = 0.051)

| Independent variable | Standardized coefficient | Standard error | *p* |
| --- | --- | --- | --- |
| Age | -0.095 | 0.322 | 0.151 |
| Sex | 0.171 | 0.272 | **0.032** |
| Kinship | 0.012 | 0.606 | 0.320 |
| Reproductive status | 0.141 | 0.318 | 0.086 |
| Left-handed gesture | 0.037 | 0.071 | 0.170 |
| Right-handed gesture | -0.028 | 0.245 | 0.470 |

Table S15.10 Rate of mating (*r^2^* = 0.254)

| Independent variable | Standardized coefficient | Standard error | *p* |
| --- | --- | --- | --- |
| Age | -0.010 | 0.179 | 0.506 |
| Sex | -0.200 | 0.158 | **0.009** |
| Kinship | -0.070 | 0.321 | 0.075 |
| Reproductive status | 0.089 | 0.162 | 0.218 |
| Left-handed gesture | -0.156 | 0.035 | **0.028** |
| Right-handed gesture | 0.505 | 0.138 | **0.005** |

Table S15.11 Rate of scratch produced (*r^2^* = 0.060)

| Independent variable | Standardized coefficient | Standard error | *p* |
| --- | --- | --- | --- |
| Age | 0.024 | 0.611 | 0.37481 |
| Sex | 0.197 | 0.557 | **0.03998** |
| Kinship | 0.079 | 1.116 | 0.18241 |
| Reproductive status | 0.004 | 0.433 | 0.47276 |
| Left-handed gesture | 0.021 | 0.115 | 0.33833 |
| Right-handed gesture | 0.108 | 0.431 | 0.14293 |

Table S15.12 Rate of scratch received (*r^2^* = 0.078)

| Independent variable | Standardized coefficient | Standard error | *p* |
| --- | --- | --- | --- |
| Age | 0.018 | 0.600 | 0.42229 |
| Sex | 0.213 | 0.566 | **0.03448** |
| Kinship | 0.074 | 1.146 | 0.1924 |
| Reproductive status | 0.002 | 0.427 | 0.50725 |
| Left-handed gesture | -0.176 | 0.120 | **0.01999** |
| Right-handed gesture | 0.196 | 0.419 | **0.04448** |

Table S15.13 Duration of proximity to 10 meters (*r^2^* = 0.068)

| Independent variable | Standardized coefficient | Standard error | *p* |
| --- | --- | --- | --- |
| Age | 0.169 | 3.672 | 0.053 |
| Sex | -0.053 | 3.036 | 0.304 |
| Kinship | 0.056 | 6.518 | 0.273 |
| Reproductive status | -0.031 | 3.161 | 0.356 |
| Left-handed gesture | -0.118 | 0.686 | 0.133 |
| Right-handed gesture | 0.253 | 2.527 | **0.007** |

Table S15.14 Rate of lipsmack produced (*r^2^* = 0.131)

| Independent variable | Standardized coefficient | Standard error | *p* |
| --- | --- | --- | --- |
| Age | 0.244 | 0.510 | 0.019 |
| Sex | 0.029 | 0.405 | 0.380 |
| Kinship | 0.010 | 0.911 | 0.290 |
| Reproductive status | 0.112 | 0.434 | 0.174 |
| Scratch produced | 0.182 | 0.072 | **0.040** |

Table S15.15 Rate of lipsmack produced (*r^2^* = 0.109)

| Independent variable | Standardized coefficient | Standard error | *p* |
| --- | --- | --- | --- |
| Age | 0.248 | 0.516 | **0.010** |
| Sex | 0.085 | 0.435 | 0.214 |
| Kinship | 0.032 | 0.857 | 0.187 |
| Reproductive status | 0.121 | 0.417 | 0.133 |
| Scratch received | -0.100 | 0.069 | **0.049** |

# Centrality and Laterality

Table S16. Node-level regression models predicting durations of social behavior, per hour dyad spent in the same party produced (outdegree). Predictors were left-handed and right-handed gestures produced (outdegree) or received (indegree). Based on 12 chimpanzees. Significant *p* values are indicated in bold.

Table S16.1. Duration of proximity to 2 meters outdegree (*r^2^* = 0.951)

| Independent variable | Standardized coefficient | *p* |
| --- | --- | --- |
| Reproductive status | -0.347 | 0.210 |
| Kinship | 0.092 | 0.426 |
| Age/ sex | 0.037 | 0.449 |
| Left-handed gesture outdegree | -0.149 | 0.416 |
| Left-handed gesture indegree | -0.213 | 0.344 |
| Right-handed gesture outdegree | 0.304 | 0.291 |
| Right-handed gesture indegree | 1.004 | **0.034** |

Table S16.2. Duration of joint feeding outdegree (*r^2^* = 0.732)

| Independent variable | Standardized coefficient | *p* |
| --- | --- | --- |
| Reproductive status | 0.401 | 0.179 |
| Kinship | 0.384 | 0.212 |
| Age/ sex | -0.456 | 0.190 |
| Left-handed gesture outdegree | -0.278 | 0.299 |
| Left-handed gesture indegree | -0.348 | 0.223 |
| Right-handed gesture outdegree | 0.678 | 0.159 |
| Right-handed gesture indegree | 0.187 | 0.306 |

Table S16.3. Duration of joint resting outdegree (*r^2^* = 0.929)

| Independent variable | Standardized coefficient | *p* |
| --- | --- | --- |
| Reproductive status | -0.486 | 0.114 |
| Kinship | -0.025 | 0.465 |
| Age/ sex | 0.094 | 0.368 |
| Left-handed gesture outdegree | -0.043 | 0.537 |
| Left-handed gesture indegree | -0.327 | 0.183 |
| Right-handed gesture outdegree | 0.079 | 0.382 |
| Right-handed gesture indegree | 1.135 | **0.033** |

Table S16.4. Duration of joint travel outdegree (*r^2^* = 0.944)

| Independent variable | Standardized coefficient | *p* |
| --- | --- | --- |
| Reproductive status | -0.251 | 0.312 |
| Kinship | 0.077 | 0.451 |
| Age/ sex | 0.232 | 0.309 |
| Left-handed gesture outdegree | -0.167 | 0.412 |
| Left-handed gesture indegree | 0.063 | 0.392 |
| Right-handed gesture outdegree | 0.206 | 0.352 |
| Right-handed gesture indegree | 0.961 | **0.032** |

Table S16.5. Duration of unidirectional grooming outdegree (*r^2^* = 0.695)

| Independent variable | Standardized coefficient | *p* |
| --- | --- | --- |
| Reproductive status | -0.220 | 0.345 |
| Kinship | -0.038 | 0.471 |
| Age/ sex | 0.104 | 0.418 |
| Left-handed gesture outdegree | -0.676 | 0.120 |
| Left-handed gesture indegree | -0.905 | **0.022** |
| Right-handed gesture outdegree | 1.161 | **0.032** |
| Right-handed gesture indegree | 0.423 | 0.183 |

Table S16.6. Duration of grooming received outdegree (*r^2^* = 0.937)

| Independent variable | Standardized coefficient | *p* |
| --- | --- | --- |
| Reproductive status | -0.500 | 0.111 |
| Kinship | 0.062 | 0.460 |
| Age/ sex | 0.058 | 0.416 |
| Left-handed gesture outdegree | 0.038 | 0.418 |
| Left-handed gesture indegree | 0.247 | 0.254 |
| Right-handed gesture outdegree | -0.027 | 0.493 |
| Right-handed gesture indegree | 0.938 | **0.049** |

Table S16.7. Duration of grooming mutual outdegree (*r^2^* = 0.875)

| Independent variable | Standardized coefficient | *p* |
| --- | --- | --- |
| Reproductive status | -0.272 | 0.298 |
| Kinship | -0.081 | 0.429 |
| Age/ sex | 0.034 | 0.450 |
| Left-handed gesture outdegree | -0.112 | 0.443 |
| Left-handed gesture indegree | -0.003 | 0.549 |
| Right-handed gesture outdegree | 0.100 | 0.417 |
| Right-handed gesture indegree | 0.995 | **0.025** |

Table S16.8. Duration of attention present outdegree (*r^2^* = 0.953)

| Independent variable | Standardized coefficient | *p* |
| --- | --- | --- |
| Reproductive status | -0.215 | 0.333 |
| Kinship | 0.073 | 0.447 |
| Age/ sex | -0.074 | 0.457 |
| Left-handed gesture outdegree | -0.259 | 0.334 |
| Left-handed gesture indegree | -0.204 | 0.357 |
| Right-handed gesture outdegree | 0.473 | 0.231 |
| Right-handed gesture indegree | 0.903 | **0.043** |

Table S16.9. Duration of attention absent outdegree (*r^2^* = 0.942)

| Independent variable | Standardized coefficient | *p* |
| --- | --- | --- |
| Reproductive status | -0.420 | 0.156 |
| Kinship | 0.102 | 0.426 |
| Age/ sex | 0.105 | 0.376 |
| Left-handed gesture outdegree | -0.079 | 0.482 |
| Left-handed gesture indegree | -0.215 | 0.327 |
| Right-handed gesture outdegree | 0.195 | 0.332 |
| Right-handed gesture indegree | 1.047 | **0.035** |

Table S16.10. Rate of mating outdegree (*r^2^* = 0.842)

| Independent variable | Standardized coefficient | *p* |
| --- | --- | --- |
| Reproductive status | 0.349 | 0.170 |
| Kinship | -0.251 | 0.295 |
| Age/ sex | 0.371 | 0.204 |
| Left-handed gesture outdegree | -0.911 | **0.035** |
| Left-handed gesture indegree | -0.868 | **0.036** |
| Right-handed gesture outdegree | 1.489 | **0.014** |
| Right-handed gesture indegree | -0.138 | 0.454 |

Table S16.10. Duration of proximity to 10 meters outdegree (*r^2^* = 0.524)

| Independent variable | Standardized coefficient | *p* |
| --- | --- | --- |
| Reproductive status | -0.038 | 0.500 |
| Kinship | 0.347 | 0.243 |
| Age/ sex | -0.253 | 0.329 |
| Left-handed gesture outdegree | -0.209 | 0.372 |
| Left-handed gesture indegree | -0.134 | 0.424 |
| Right-handed gesture outdegree | 0.346 | 0.298 |
| Right-handed gesture indegree | 0.357 | 0.205 |

# Communicative Complexity

Table S17. MRQAP regression models predicting indices of communicative complexity per hour dyad spent within 10 meters. Predictors were left-handed and right-handed gestures. Dyads were classified as same age or different age (within 5 years), same sex or different sex, related by maternal kinship and as the same or different reproductive status (reproductively active, not reproductively active). Based on 132 dyadic relationships of the chimpanzees. Significant *p* values are indicated in bold.

Table S17.1 Rate of bodily gestures (*r^2^* = 0.194)

| Independent variable | Standardized coefficient | Standard error | *p* |
| --- | --- | --- | --- |
| Age | 0.220 | 1.639 | **0.017** |
| Sex | -0.001 | 1.353 | 0.501 |
| Kinship | -0.024 | 3.003 | 0.417 |
| Reproductive status | 0.079 | 1.423 | 0.223 |
| Left-handed gesture | 0.326 | 0.307 | **0.008** |
| Right-handed gesture | 0.055 | 1.097 | 0.206 |

Table S17.2 Rate of manual gestures (*r^2^* = 0.751)

| Independent variable | Standardized coefficient | Standard error | *p* |
| --- | --- | --- | --- |
| Age | 0.018 | 0.380 | 0.315 |
| Sex | -0.048 | 0.329 | 0.183 |
| Kinship | -0.026 | 0.728 | 0.224 |
| Reproductive status | -0.004 | 0.313 | 0.472 |
| Left-handed gesture | 0.810 | 0.154 | **0.001** |
| Right-handed gesture | 0.103 | 0.295 | **0.047** |

Table S17.3 Rate of combined gestures with other gesture types (*r^2^* = 0.116)

| Independent variable | Standardized coefficient | Standard error | *p* |
| --- | --- | --- | --- |
| Age | 0.109 | 0.392 | 0.127 |
| Sex | -0.078 | 0.342 | 0.199 |
| Kinship | -0.038 | 0.704 | 0.333 |
| Reproductive status | 0.067 | 0.333 | 0.216 |
| Left-handed gesture | 0.207 | 0.080 | **0.042** |
| Right-handed gesture | 0.148 | 0.282 | 0.072 |

Table S17.4 Rate of non-combined gestures with other gesture types (*r^2^* = 0.451)

| Independent variable | Standardized coefficient | Standard error | *p* |
| --- | --- | --- | --- |
| Age | 0.191 | 1.302 | **0.013** |
| Sex | 0.002 | 1.060 | 0.498 |
| Kinship | -0.017 | 2.315 | 0.477 |
| Reproductive status | 0.038 | 1.073 | 0.331 |
| Left-handed gesture | 0.619 | 0.332 | **0.001** |
| Right-handed gesture | 0.034 | 0.900 | 0.260 |

Table S17.5 Rate of events (*r^2^* = 0.408)

| Independent variable | Standardized coefficient | Standard error | *p* |
| --- | --- | --- | --- |
| Age | 0.173 | 1.616 | **0.024** |
| Sex | -0.022 | 1.287 | 0.376 |
| Kinship | -0.031 | 2.817 | 0.341 |
| Reproductive status | 0.059 | 1.288 | 0.233 |
| Left-handed gesture | 0.560 | 0.374 | **0.007** |
| Right-handed gesture | 0.081 | 1.130 | 0.132 |

Table S17.6 Rate of gestures with objects (*r^2^* = 0.063)

| Independent variable | Standardized coefficient | Standard error | *p* |
| --- | --- | --- | --- |
| Age | 0.060 | 0.743 | 0.259 |
| Sex | -0.069 | 0.649 | 0.265 |
| Kinship | -0.063 | 1.319 | 0.155 |
| Reproductive status | -0.038 | 0.594 | 0.353 |
| Left-handed gesture | 0.192 | 0.163 | **0.044** |
| Right-handed gesture | 0.076 | 0.549 | 0.141 |

Table S17.7 Rate of gestures without objects (*r^2^* = 0.442)

| Independent variable | Standardized coefficient | Standard error | *p* |
| --- | --- | --- | --- |
| Age | 0.187 | 1.429 | **0.015** |
| Sex | 0.007 | 1.131 | 0.433 |
| Kinship | -0.007 | 2.392 | 0.486 |
| Reproductive status | 0.087 | 1.325 | 0.186 |
| Left-handed gesture | 0.577 | 0.358 | **0.009** |
| Right-handed gesture | 0.072 | 1.020 | 0.131 |

Table S17.8 Rate of indicative gestures (*r^2^* = 0.126)

| Independent variable | Standardized coefficient | Standard error | *p* |
| --- | --- | --- | --- |
| Age | 0.150 | 0.121 | **0.049** |
| Sex | -0.034 | 0.096 | 0.357 |
| Kinship | 0.001 | 0.212 | 0.395 |
| Reproductive status | 0.086 | 0.111 | 0.181 |
| Left-handed gesture | 0.160 | 0.028 | **0.043** |
| Right-handed gesture | 0.186 | 0.098 | **0.042** |

Table S17.9 Rate of non-indicative gestures (*r^2^* = 0.758)

| Independent variable | Standardized coefficient | Standard error | *p* |
| --- | --- | --- | --- |
| Age | -0.007 | 0.350 | 0.488 |
| Sex | -0.045 | 0.304 | 0.188 |
| Kinship | -0.029 | 0.680 | 0.177 |
| Reproductive status | -0.022 | 0.283 | 0.337 |
| Left-handed gesture | 0.830 | 0.152 | **0.001** |
| Right-handed gesture | 0.078 | 0.294 | 0.076 |

Table S17.10 Rate of unimodal gestures (*r^2^* = 0.518)

| Independent variable | Standardized coefficient | Standard error | *p* |
| --- | --- | --- | --- |
| Age | 0.163 | 0.986 | **0.010** |
| Sex | -0.025 | 0.811 | 0.362 |
| Kinship | -0.004 | 1.751 | 0.577 |
| Reproductive status | 0.090 | 0.829 | 0.100 |
| Left-handed gesture | 0.616 | 0.244 | **0.004** |
| Right-handed gesture | 0.120 | 0.752 | 0.070 |

Table S17.11 Rate of multimodal gesture (high amplitude vocalization) (*r^2^* = 0.078)

| Independent variable | Standardized coefficient | Standard error | *p* |
| --- | --- | --- | --- |
| Age | 0.106 | 0.819 | 0.154 |
| Sex | 0.045 | 0.701 | 0.304 |
| Kinship | -0.028 | 1.516 | 0.464 |
| Reproductive status | -0.036 | 0.672 | 0.342 |
| Left-handed gesture | 0.277 | 0.151 | **0.025** |
| Right-handed gesture | -0.158 | 0.564 | **0.011** |

Table S17.12 Rate of multimodal gesture (low amplitude vocalization) (*r^2^* = 0.053)

| Independent variable | Standardized coefficient | Standard error | *p* |
| --- | --- | --- | --- |
| Age | 0.067 | 0.203 | 0.213 |
| Sex | -0.018 | 0.164 | 0.448 |
| Kinship | -0.049 | 0.356 | 0.280 |
| Reproductive status | 0.023 | 0.177 | 0.446 |
| Left-handed gesture | -0.080 | 0.050 | 0.175 |
| Right-handed gesture | 0.242 | 0.177 | **0.046** |

Table S17.13 Rate of multimodal gesture (facial expression) (*r^2^* = 0.104)

| Independent variable | Standardized coefficient | Standard error | *p* |
| --- | --- | --- | --- |
| Age | 0.058 | 0.131 | 0.270 |
| Sex | 0.068 | 0.108 | 0.237 |
| Kinship | 0.007 | 0.239 | 0.273 |
| Reproductive status | 0.112 | 0.117 | 0.157 |
| Left-handed gesture | 0.303 | 0.027 | **0.023** |
| Right-handed gesture | -0.087 | 0.086 | 0.091 |

Table S17.14 Rate of gestures accompanied by visual attention present (*r^2^* = 0.195)

| Independent variable | Standardized coefficient | Standard error | *p* |
| --- | --- | --- | --- |
| Age | 0.119 | 0.949 | 0.115 |
| Sex | 0.042 | 0.756 | 0.310 |
| Kinship | -0.007 | 1.680 | 0.593 |
| Reproductive status | 0.074 | 0.860 | 0.232 |
| Left-handed gesture | 0.327 | 0.196 | **0.016** |
| Right-handed gesture | 0.107 | 0.687 | 0.105 |

Table S17.15 Rate of gestures unaccompanied by visual attention (*r^2^* = 0.556)

| Independent variable | Standardized coefficient | Standard error | *p* |
| --- | --- | --- | --- |
| Age | 0.116 | 0.558 | 0.054 |
| Sex | 0.051 | 0.458 | 0.231 |
| Kinship | 0.021 | 1.029 | 0.217 |
| Reproductive status | 0.074 | 0.438 | 0.123 |
| Left-handed gesture | 0.692 | 0.163 | **0.001** |
| Right-handed gesture | 0.041 | 0.407 | 0.145 |

Table S17.16 Rate of gestures accompanied by penile erection (*r^2^* = 0.214)

| Independent variable | Standardized coefficient | Standard error | *p* |
| --- | --- | --- | --- |
| Age | -0.020 | 0.219 | 0.455 |
| Sex | -0.176 | 0.189 | **0.015** |
| Kinship | -0.074 | 0.394 | 0.079 |
| Reproductive status | 0.023 | 0.180 | 0.414 |
| Left-handed gesture | -0.046 | 0.049 | 0.195 |
| Right-handed gesture | 0.446 | 0.178 | **0.006** |

Table S17.17 Rate of gestures accompanied by piloerection (*r^2^* = 0.122)

| Independent variable | Standardized coefficient | Standard error | *p* |
| --- | --- | --- | --- |
| Age | 0.169 | 1.307 | 0.059 |
| Sex | -0.070 | 1.102 | 0.234 |
| Kinship | -0.029 | 2.256 | 0.449 |
| Reproductive status | 0.032 | 1.008 | 0.365 |
| Left-handed gesture | 0.349 | 0.251 | **0.016** |
| Right-handed gesture | -0.082 | 0.881 | 0.123 |

Table S17.18 Rate of gestures produced singly (*r^2^* = 0.668)

| Independent variable | Standardized coefficient | Standard error | *p* |
| --- | --- | --- | --- |
| Age | 0.182 | 0.623 | **0.003** |
| Sex | 0.022 | 0.486 | 0.348 |
| Kinship | 0.017 | 1.039 | 0.281 |
| Reproductive status | 0.107 | 0.569 | 0.052 |
| Left-handed gesture | 0.704 | 0.183 | **0.001** |
| Right-handed gesture | 0.116 | 0.412 | **0.043** |

Table S17.19 Rate of rapid sequence (*r^2^* = 0.124)

| Independent variable | Standardized coefficient | Standard error | *p* |
| --- | --- | --- | --- |
| Age | 0.204 | 0.338 | **0.030** |
| Sex | -0.086 | 0.281 | 0.211 |
| Kinship | -0.049 | 0.608 | 0.304 |
| Reproductive status | 0.011 | 0.274 | 0.456 |
| Left-handed gesture | 0.327 | 0.062 | **0.016** |
| Right-handed gesture | -0.057 | 0.220 | 0.266 |

Table S17.20 Rate of persistence sequence (*r^2^* = 0.095)

| Independent variable | Standardized coefficient | Standard error | *p* |
| --- | --- | --- | --- |
| Age | -0.043 | 0.100 | 0.342 |
| Sex | 0.122 | 0.084 | 0.100 |
| Kinship | -0.031 | 0.180 | 0.362 |
| Reproductive status | -0.011 | 0.084 | 0.455 |
| Left-handed gesture | 0.011 | 0.022 | 0.305 |
| Right-handed gesture | 0.274 | 0.078 | **0.028** |

Table S17.21 Rate of dyadic repertoire size (*r^2^* = 0.273)

| Independent variable | Standardized coefficient | Standard error | *p* |
| --- | --- | --- | --- |
| Age | 0.122 | 1.139 | 0.096 |
| Sex | -0.024 | 0.948 | 0.402 |
| Kinship | -0.038 | 2.087 | 0.321 |
| Reproductive status | 0.023 | 0.956 | 0.401 |
| Left-handed gesture | 0.515 | 0.249 | **0.011** |
| Right-handed gesture | -0.017 | 0.835 | 0.496 |

Table S17.22 Rate of repetitive gestures (*r^2^* = 0.368)

| Independent variable | Standardized coefficient | Standard error | *p* |
| --- | --- | --- | --- |
| Age | 0.159 | 1.036 | **0.040** |
| Sex | 0.004 | 0.857 | 0.477 |
| Kinship | -0.029 | 1.850 | 0.436 |
| Reproductive status | 0.003 | 0.828 | 0.490 |
| Left-handed gesture | 0.500 | 0.229 | **0.003** |
| Right-handed gesture | 0.135 | 0.727 | 0.087 |

Table S17.23 Rate of non-repetitive gestures (*r^2^* = 0.212)

| Independent variable | Standardized coefficient | Standard error | *p* |
| --- | --- | --- | --- |
| Age | 0.147 | 1.404 | 0.092 |
| Sex | -0.042 | 1.157 | 0.347 |
| Kinship | 0.014 | 2.425 | 0.283 |
| Reproductive status | 0.094 | 1.194 | 0.185 |
| Left-handed gesture | 0.418 | 0.266 | **0.010** |
| Right-handed gesture | 0.014 | 0.905 | 0.315 |

Table S17.24 Rate of homogeneous gestures (*r^2^* = 0.283)

| Independent variable | Standardized coefficient | Standard error | *p* |
| --- | --- | --- | --- |
| Age | 0.259 | 1.270 | **0.003** |
| Sex | 0.043 | 1.034 | 0.304 |
| Kinship | 0.008 | 2.137 | 0.396 |
| Reproductive status | 0.097 | 1.054 | 0.153 |
| Left-handed gesture | 0.441 | 0.262 | **0.007** |
| Right-handed gesture | -0.032 | 0.850 | 0.370 |

Table S17.25 Rate of heterogeneous gestures (*r^2^* = 0.502)

| Independent variable | Standardized coefficient | Standard error | *p* |
| --- | --- | --- | --- |
| Age | -0.067 | 0.452 | 0.172 |
| Sex | -0.054 | 0.380 | 0.220 |
| Kinship | -0.054 | 0.834 | 0.107 |
| Reproductive status | -0.083 | 0.382 | 0.142 |
| Left-handed gesture | 0.625 | 0.131 | **0.003** |
| Right-handed gesture | 0.137 | 0.329 | **0.043** |

Table S17.26 Rate of close proximity gestures (*r^2^* = 0.588)

| Independent variable | Standardized coefficient | Standard error | *p* |
| --- | --- | --- | --- |
| Age | 0.157 | 0.726 | **0.008** |
| Sex | 0.067 | 0.579 | 0.144 |
| Kinship | 0.033 | 1.268 | 0.182 |
| Reproductive status | 0.092 | 0.646 | 0.125 |
| Left-handed gesture | 0.699 | 0.209 | **0.001** |
| Right-handed gesture | 0.038 | 0.527 | 0.188 |

Table S17.27 Rate of far proximity gestures (*r^2^* = 0.101)

| Independent variable | Standardized coefficient | Standard error | *p* |
| --- | --- | --- | --- |
| Age | 0.117 | 1.304 | 0.144 |
| Sex | -0.035 | 1.085 | 0.368 |
| Kinship | -0.050 | 2.250 | 0.274 |
| Reproductive status | -0.006 | 1.051 | 0.481 |
| Left-handed gesture | 0.317 | 0.243 | **0.016** |
| Right-handed gesture | -0.043 | 0.856 | 0.358 |

Table S17.28 Rate of auditory long-range gestures (*r^2^* = 0.052)

| Independent variable | Standardized coefficient | Standard error | *p* |
| --- | --- | --- | --- |
| Age | 0.058 | 0.550 | 0.266 |
| Sex | -0.058 | 0.472 | 0.292 |
| Kinship | -0.067 | 0.982 | 0.133 |
| Reproductive status | -0.045 | 0.456 | 0.334 |
| Left-handed gesture | 0.101 | 0.117 | 0.102 |
| Right-handed gesture | 0.149 | 0.435 | 0.098 |

Table S17.29 Rate of auditory short-range gestures (*r^2^* = 0.138)

| Independent variable | Standardized coefficient | Standard error | *p* |
| --- | --- | --- | --- |
| Age | 0.250 | 0.583 | **0.008** |
| Sex | 0.037 | 0.478 | 0.334 |
| Kinship | 0.020 | 0.999 | 0.184 |
| Reproductive status | 0.070 | 0.475 | 0.282 |
| Left-handed gesture | 0.113 | 0.105 | **0.049** |
| Right-handed gesture | 0.152 | 0.422 | 0.074 |

Table S17.30 Rate of tactile gestures (*r^2^* = 0.806)

| Independent variable | Standardized coefficient | Standard error | *p* |
| --- | --- | --- | --- |
| Age | 0.048 | 0.296 | 0.186 |
| Sex | 0.046 | 0.251 | 0.193 |
| Kinship | 0.024 | 0.545 | 0.121 |
| Reproductive status | 0.043 | 0.249 | 0.232 |
| Left-handed gesture | 0.901 | 0.112 | **0.001** |
| Right-handed gesture | -0.028 | 0.199 | 0.195 |

Table S17.31 Rate of visual gestures (*r^2^* = 0.188)

| Independent variable | Standardized coefficient | Standard error | *p* |
| --- | --- | --- | --- |
| Age | 0.143 | 1.347 | 0.079 |
| Sex | -0.061 | 1.087 | 0.235 |
| Kinship | -0.041 | 2.454 | 0.314 |
| Reproductive status | 0.049 | 1.153 | 0.340 |
| Left-handed gesture | 0.399 | 0.299 | **0.020** |
| Right-handed gesture | 0.015 | 0.943 | 0.334 |

Table S17.32 Rate of response present (*r^2^* = 0.432)

| Independent variable | Standardized coefficient | Standard error | *p* |
| --- | --- | --- | --- |
| Age | 0.112 | 0.729 | 0.121 |
| Sex | 0.019 | 0.591 | 0.374 |
| Kinship | 0.012 | 1.341 | 0.265 |
| Reproductive status | 0.083 | 0.643 | 0.171 |
| Left-handed gesture | 0.683 | 0.184 | **0.009** |
| Right-handed gesture | -0.107 | 0.497 | **0.031** |

Table S17.33 Rate of response absent (*r^2^* = 0.364)

| Independent variable | Standardized coefficient | Standard error | *p* |
| --- | --- | --- | --- |
| Age | 0.179 | 0.522 | **0.028** |
| Sex | 0.049 | 0.448 | 0.283 |
| Kinship | 0.007 | 0.924 | 0.343 |
| Reproductive status | 0.060 | 0.427 | 0.266 |
| Left-handed gesture | 0.372 | 0.110 | **0.010** |
| Right-handed gesture | 0.253 | 0.357 | **0.028** |

Table S17.34 Rate of repetition (*r^2^* = 0.040)

| Independent variable | Standardized coefficient | Standard error | *p* |
| --- | --- | --- | --- |
| Age | 0.115 | 0.060 | 0.134 |
| Sex | 0.009 | 0.051 | 0.439 |
| Kinship | -0.015 | 0.107 | 0.571 |
| Reproductive status | -0.024 | 0.044 | 0.414 |
| Left-handed gesture | -0.048 | 0.011 | 0.148 |
| Right-handed gesture | 0.189 | 0.046 | 0.065 |

Table S17.35 Rate of elaboration (*r^2^* = 0.101)

| Independent variable | Standardized coefficient | Standard error | *p* |
| --- | --- | --- | --- |
| Age | -0.115 | 0.116 | 0.084 |
| Sex | 0.087 | 0.102 | 0.165 |
| Kinship | -0.055 | 0.206 | 0.209 |
| Reproductive status | 0.040 | 0.102 | 0.304 |
| Left-handed gesture | 0.030 | 0.026 | 0.237 |
| Right-handed gesture | 0.253 | 0.095 | **0.032** |

Table S18. MRQAP regression models predicting rate of gestures produced across functions per hour dyad spent within 10 meters. Predictors were right and left handed gestures. Dyads were classified as same age or different age (within 5 years), same sex or different sex, related by maternal kinship and as the same or different reproductive status (reproductively active, not reproductively active). Based on 132 dyadic relationships of the chimpanzees. Significant *p* values are indicated in bold.

Table S18.1 Rate of Copulation gestures (*r^2^* = 0.254)

| Independent variable | Standardized coefficient | Standard error | *p* |
| --- | --- | --- | --- |
| Age | -0.010 | 0.178 | 0.52124 |
| Sex | -0.200 | 0.158 | **0.005** |
| Kinship | -0.070 | 0.317 | 0.08846 |
| Reproductive status | 0.089 | 0.157 | 0.22289 |
| Left-handed gesture | -0.156 | 0.037 | **0.03048** |
| Right-handed gesture | 0.505 | 0.149 | **0.0045** |

Table S18.2 Rate of Threat to dominate gestures (*r^2^* = 0.139)

| Independent variable | Standardized coefficient | Standard error | *p* |
| --- | --- | --- | --- |
| Age | 0.140 | 0.168 | 0.133 |
| Sex | -0.020 | 0.143 | 0.464 |
| Kinship | 0.012 | 0.280 | 0.247 |
| Reproductive status | 0.099 | 0.132 | 0.146 |
| Left-handed gesture | 0.396 | 0.030 | **0.012** |
| Right-handed gesture | -0.235 | 0.113 | **0.006** |

Table S18.3 Rate of Give groom gestures (*r^2^* = 0.179)

| Independent variable | Standardized coefficient | Standard error | *p* |
| --- | --- | --- | --- |
| Age | 0.242 | 0.510 | **0.01649** |
| Sex | 0.040 | 0.405 | 0.32834 |
| Kinship | 0.021 | 0.890 | 0.1929 |
| Reproductive status | 0.069 | 0.410 | 0.30735 |
| Left-handed gesture | 0.160 | 0.089 | **0.03798** |
| Right-handed gesture | 0.196 | 0.318 | **0.04898** |

Table S18.4 Rate of Mutually groom gestures (*r^2^* = 0.150)

| Independent variable | Standardized coefficient | Standard error | *p* |
| --- | --- | --- | --- |
| Age | 0.153 | 0.166 | 0.10645 |
| Sex | -0.005 | 0.138 | 0.53073 |
| Kinship | 0.018 | 0.312 | 0.24238 |
| Reproductive status | 0.091 | 0.130 | 0.17791 |
| Left-handed gesture | 0.406 | 0.030 | **0.01049** |
| Right-handed gesture | -0.237 | 0.116 | **0.003** |

Table S18.5 Rate of Receive groom gestures (*r^2^* = 0.206)

| Independent variable | Standardized coefficient | Standard error | *p* |
| --- | --- | --- | --- |
| Age | 0.062 | 0.190 | 0.263 |
| Sex | 0.103 | 0.162 | 0.144 |
| Kinship | 0.046 | 0.348 | 0.172 |
| Reproductive status | 0.117 | 0.186 | 0.152 |
| Left-handed gesture | 0.439 | 0.043 | **0.011** |
| Right-handed gesture | -0.096 | 0.131 | 0.065 |

Table S18.6 Rate of Other threat gestures (*r^2^* = 0.044)

| Independent variable | Standardized coefficient | Standard error | *p* |
| --- | --- | --- | --- |
| Age | -0.039 | 0.088 | 0.376 |
| Sex | 0.145 | 0.078 | 0.089 |
| Kinship | -0.034 | 0.155 | 0.406 |
| Reproductive status | -0.107 | 0.069 | 0.133 |
| Left-handed gesture | -0.119 | 0.018 | **0.034** |
| Right-handed gesture | 0.187 | 0.065 | **0.049** |

Table S18.7 Rate of Synchronized high-intensity panthoot gestures (*r^2^* = 0.045)

| Independent variable | Standardized coefficient | Standard error | *p* |
| --- | --- | --- | --- |
| Age | 0.195 | 0.271 | 0.054 |
| Sex | -0.021 | 0.228 | 0.477 |
| Kinship | -0.018 | 0.512 | 0.543 |
| Reproductive status | 0.022 | 0.208 | 0.451 |
| Left-handed gesture | 0.105 | 0.042 | **0.049** |
| Right-handed gesture | -0.063 | 0.182 | 0.215 |

Table S18.8 Rate of Solo high-intensity panthoot gestures (*r^2^* = 0.042)

| Independent variable | Standardized coefficient | Standard error | *p* |
| --- | --- | --- | --- |
| Age | 0.001 | 0.112 | 0.43628 |
| Sex | -0.037 | 0.094 | 0.4013 |
| Kinship | -0.044 | 0.202 | 0.23138 |
| Reproductive status | -0.061 | 0.098 | 0.35632 |
| Left-handed gesture | 0.222 | 0.024 | **0.03198** |
| Right-handed gesture | -0.098 | 0.087 | **0.04948** |

Table S18.9 Rate of Synchronized low-intensity panthoot gestures (*r^2^* = 0.015)

| Independent variable | Standardized coefficient | Standard error | *p* |
| --- | --- | --- | --- |
| Age | 0.024 | 0.090 | 0.29835 |
| Sex | -0.052 | 0.075 | 0.36732 |
| Kinship | -0.047 | 0.169 | 0.18491 |
| Reproductive status | -0.072 | 0.073 | 0.34583 |
| Left-handed gesture | -0.063 | 0.016 | 0.07396 |
| Right-handed gesture | 0.107 | 0.065 | 0.07546 |

Table S18.10 Rate of Greeting gestures (*r^2^* = 0.059)

| Independent variable | Standardized coefficient | Standard error | *p* |
| --- | --- | --- | --- |
| Age | 0.104 | 0.166 | 0.12544 |
| Sex | -0.042 | 0.126 | 0.30085 |
| Kinship | -0.031 | 0.293 | 0.43328 |
| Reproductive status | 0.086 | 0.172 | 0.22339 |
| Left-handed gesture | 0.036 | 0.043 | 0.21739 |
| Right-handed gesture | 0.167 | 0.158 | 0.11594 |

**References**

Bakeman, R., & Gottman, J. M. (1997). *Observing Interaction: An Introduction to Sequential Analysis*. New York: Cambridge University Press.

Bard, K. A. (1992). Intentional Behavior and Intentional Communication in Young Free-Ranging Orangutans. *Child Development, 63*(5), 1186-1197.

Borgatti, S. P., Everett, M. G., & Johnson, J. C. (2013). *Analyzing Social Networks*: SAGE Publications Limited.

Hopkins, W. D., Fernandez-Carriba, S., Micheal, W., J., Hostetter, A., Pilcher, D., & Poss, S. (2001). The use of bouts and frequencies in the evaluation of hand preferences for a coordinated bimanual task in chimpanzees (*Pan troglodytes*): An empirical study comparing two different indicies of laterality. *Journal of Comparative Psychology, 115*(3), 294-299.

Mitani, J. C. (2009). Male chimpanzees form enduring and equitable social bonds. *Animal Behaviour, 77*(3), 633-640.

Roberts, A. I., & Roberts, S. G. B. (in press). Gesture sequences are associated with social complexity in wild chimpanzees. *Animal Cognition*.

Roberts, A. I., Roberts, S. G. B., & Vick, S.-J. (2014). The repertoire and intentionality of gestural communication in wild chimpanzees. *Animal Cognition, 17*(2), 317 - 336. doi: 10.1007/s10071-013-0664-5

Roberts, A. I., Vick, S.-J., Roberts, S. G. B., Buchanan-Smith, H. M., & Zuberbühler, K. (2012). A structure-based repertoire of manual gestures in wild chimpanzees: Statistical analyses of a graded communication system. *Evolution and Human Behavior, 33*(5), 578-589. doi: 10.1016/j.evolhumbehav.2012.05.006

Roberts, S. G. B., & Roberts, A. I. (2016). Social brain hypothesis, vocal and gesture networks of wild chimpanzees. *Frontiers in Psychology, 7*(1756). doi: 10.3389/fpsyg.2016.01756
