# Supplementary material for: Complex Sociality of Wild Chimpanzees Can Emerge from Laterality of Manual Gestures
Source: Hum Nat. 2019 Jun 24;30(3):299–325. doi: 10.1007/s12110-019-09347-3 (PMC6698263; doi:10.1007/s12110-019-09347-3)
Supplement: Supplementary file 5 — (DOCX 14 kb) [file 12110_2019_9347_MOESM3_ESM.docx]

Electronic Supplementary Material (ESM) - 3

for

Complex Sociality of Wild Chimpanzees Can Emerge from Laterality of Manual Gestures

Anna Ilona Roberts, Lindsay Murray, Sam George Bradley Roberts

*Human Nature* 30(3), 2019. Doi: to be added in proofs.

**Summary of results**

Multiple Regression Quadratic Assignment Procedure (MRQAP) regression models predicting dependent variables: rate of mating, scratch and duration of social behaviour from rates of right-handed and left-handed gestural communication produced per hour spent within 10 meters of the recipient. Summary table provides standardized coefficients (standard errors) and *p* values. In all models, the dependent variable was the duration of behaviour in mins, per hour dyad spent in the same party or rate of scratch or mating per hour spent within 10 meters. Green shading indicates statistically significant positive relationships, red shading indicates statistically significant negative relationships. Full results for all models are provided in Supplementary Tables.

|  | Joint activity | | | Grooming | | | Attention | | Proximity | | Mating | Scratch | |
| --- | --- | --- | --- | --- | --- | --- | --- | --- | --- | --- | --- | --- | --- |
|  | Feed | Rest | Travel | Give | Mutual | Receive | Present | Absent | To 2 meters | To 10 meters |  | Produced | Received |
| Age | 0.026 (0.326) | 0.276 (1.295)** | 0.261 (0.253)* | 0.258 (0.244)** | 0.215 (0.462)* | -0.095 (0.322) | 0.160 (1.002) | 0.312 (1.461)** | 0.318 (1.895)** | 0.169 (3.672) | -0.010 (0.179) | 0.024 (0.611) | 0.018 (0.600) |
| Sex | 0.088 (0.295) | -0.160 (1.067)* | 0.025 (0.209) | 0.030 (0.190) | -0.049 (0.393) | 0.171 (0.272)* | 0.054 (0.774) | -0.114 (1.146) | -0.061 (1.438) | -0.053 (3.036) | -0.200 (0.158)** | 0.197 (0.557)* | 0.213 (0.566)* |
| Kinship | 0.287 (0.662)* | -0.025 (2.191) | 0.009 (0.475) | 0.152 (0.444) | 0.042 (0.842) | 0.012 (0.606) | 0.178 (1.828) | 0.039 (2.512) | 0.118 (3.259) | 0.056 (6.518) | -0.070 (0.321) | 0.079 (1.116) | 0.074 (1.146) |
| Reproductive status | 0.225 (0.288)** | -0.072 (1.032) | 0.017 (0.203) | 0.083 (0.219) | 0.117 (0.410) | 0.141 (0.318) | 0.223 (0.967)* | -0.034 (1.137) | 0.084 (1.806) | -0.031 (3.161) | 0.089 (0.162) | 0.004 (0.433) | 0.002 (0.427) |
| Left-handed | -0.102 (0.063) | -0.009 (0.244) | 0.205 (0.043)* | 0.153 (0.051) | 0.241 (0.081)* | 0.037 (0.071) | 0.204 (0.155)* | -0.005 (0.260) | 0.097 (0.344) | -0.118 (0.686) | -0.156 (0.035)* | 0.021 (0.115) | -0.176 (0.120)* |
| Right-handed | 0.071 (0.235) | 0.060 (0.948) | 0.011 (0.146) | 0.278 (0.182)* | -0.003 (0.265) | -0.028 (0.245) | 0.026 (0.588) | 0.099 (1.032) | 0.089 (1.247) | 0.253  (2.527)** | 0.505 (0.138)** | 0.108 (0.431) | 0.196 (0.419)* |

* *p* < 0.05, ** *p* < 0.01, *** *p* < 0.001
