# Supplementary material for: Complex Sociality of Wild Chimpanzees Can Emerge from Laterality of Manual Gestures
Source: Hum Nat. 2019 Jun 24;30(3):299–325. doi: 10.1007/s12110-019-09347-3 (PMC6698263; doi:10.1007/s12110-019-09347-3)
Supplement: Supplementary file 6 — (DOCX 13 kb) [file 12110_2019_9347_MOESM4_ESM.docx]

Electronic Supplementary Material (ESM) - 4

for

Complex Sociality of Wild Chimpanzees Can Emerge from Laterality of Manual Gestures

Anna Ilona Roberts, Lindsay Murray, Sam George Bradley Roberts

*Human Nature* 30(3), 2019. Doi: to be added in proofs.

**Summary of results**

Node-level regression models predicting rate of mating and duration of social behaviour out degree from rates of right-handed and left-handed gestural communication in and out degree. Summary table provides standardized coefficients and *p* values. In all models, the dependent variable was the duration of behaviour in mins, per hour dyad spent in the same party out degree. Green shading indicates statistically significant positive relationships, red shading indicates statistically significant negative relationships. Full results for all models are provided in Supplementary Tables.

| Behaviour | Joint activity out degree | | | Groom out degree | | | Attention out degree | | Proximity | | Mating out degree |
| --- | --- | --- | --- | --- | --- | --- | --- | --- | --- | --- | --- |
|  | Feed | Rest | Travel | Give | Mutual | Receive | Present | absent | To 2 meters | To 10 meters |  |
| Left-handed out degree | -0.278 | -0.043 | -0.167 | -0.676 | -0.112 | 0.038 | -0.259 | -0.079 | -0.149 | -0.209 | -0.911  * |
| Left-handed in degree | -0.348 | -0.327 | 0.063 | -0.905  * | -0.003 | 0.247 | -0.204 | -0.215 | -0.213 | -0.134 | -0.868  * |
| Right-handed out degree | 0.678 | 0.079 | 0.206 | 1.161  * | 0.100 | -0.027 | 0.473 | 0.195 | 0.304 | 0.346 | 1.489  * |
| Right-handed in degree | 0.187 | 1.135  * | 0.961  * | 0.423 | 0.995  * | 0.938  * | 0.903  * | 1.047  * | 1.004  * | 0.357 | -0.138 |

* *p* < 0.05, ** *p* < 0.01, *** *p* < 0.001
