# Supplementary material for: Complex Sociality of Wild Chimpanzees Can Emerge from Laterality of Manual Gestures
Source: Hum Nat. 2019 Jun 24;30(3):299–325. doi: 10.1007/s12110-019-09347-3 (PMC6698263; doi:10.1007/s12110-019-09347-3)
Supplement: Supplementary file 7 — (DOCX 17 kb) [file 12110_2019_9347_MOESM5_ESM.docx]

Electronic Supplementary Material (ESM) - 5

for

Complex Sociality of Wild Chimpanzees Can Emerge from Laterality of Manual Gestures

Anna Ilona Roberts, Lindsay Murray, Sam George Bradley Roberts

*Human Nature* 30(3), 2019. Doi: to be added in proofs.

**Summary of results**

Multiple Regression Quadratic Assignment Procedure (MRQAP) regression models predicting rate of gestures categorized according to presence of morphological components from rates of right-handed and left-handed gestural communication. Summary table provides standardized coefficients (standard errors) and *p* values. In all models, the dependent variable was the rate of communication per hour dyad spent within 10 meters. Green shading indicates statistically significant positive relationships, red shading indicates statistically significant negative relationships. Grey shading indicates a different set of models. Full results for all models are provided in Supplementary Tables.

| *Gesture* | Bodily | Manual | Unimodal | High ampli-tude call | Low ampli-tude call | Homo-geneous | Hetero-geneous | Indica-tive | Non-indica-tive | Visual | Tactile | Audi-tory short-range | Audi-tory long-range | Single | Rapid | Persis-tence |
| --- | --- | --- | --- | --- | --- | --- | --- | --- | --- | --- | --- | --- | --- | --- | --- | --- |
| Age | 0.220 (1.639)* | 0.018 (0.380) | 0.163 (0.986)* | 0.106 (0.819) | 0.067 (0.203) | 0.259 (1.270) | -0.067 (0.452) | 0.150 (0.121)* | -0.007 (0.350) | 0.143 (1.347) | 0.048 (0.296) | 0.250 (0.583) ** | 0.058 (0.550) | 0.182 (0.623)** | 0.204 (0.338)* | -0.043 (0.100) |
| Sex | -0.001 (1.353) | -0.048 (0.329) | -0.025 (0.811) | 0.045 (0.701) | -0.018 (0.164) | 0.043 (1.034) | -0.054 (0.380) | -0.034 (0.096) | -0.045 (0.304) | -0.061 (1.087) | 0.046 (0.251) | 0.037 (0.478) | -0.058 (0.472) | 0.022 (0.486) | -0.086 (0.281) | 0.122 (0.084) |
| Kinship | -0.024 (3.003) | -0.026 (0.728) | -0.004 (1.751) | -0.028 (1.516) | -0.049 (0.356) | 0.008 (2.137) | -0.054 (0.834) | 0.001 (0.212) | -0.029 (0.680) | -0.041 (2.454) | 0.024 (0.545) | 0.020 (0.999) | -0.067 (0.982) | 0.017 (1.039) | -0.049 (0.608) | -0.031 (0.180) |
| Reproductive status | 0.079 (1.423) | -0.004 (0.313) | 0.090 (0.829) | -0.036 (0.672) | 0.023 (0.177) | 0.097 (1.054) | -0.083 (0.382) | 0.086 (0.111) | -0.022 (0.283) | 0.049 (1.153) | 0.043 (0.249) | 0.070 (0.475) | -0.045 (0.456) | 0.107 (0.569) | 0.011 (0.274) | -0.011 (0.084) |
| Left-handed | 0.326 (0.307) ** | 0.810 (0.154)** | 0.616 (0.244)** | 0.277 (0.151)* | -0.080 (0.050) | 0.441 (0.262) ** | 0.625 (0.131) ** | 0.160 (0.028)* | 0.830 (0.152) ** | 0.399 (0.299)* | 0.901 (0.112) ** | 0.113 (0.105)* | 0.101 (0.117) | 0.704 (0.183)** | 0.327 (0.062)* | 0.011 (0.022) |
| Right-handed | 0.055 (1.097) | 0.103 (0.295)* | 0.120 (0.752) | -0.158 (0.564)* | 0.242 (0.177)* | -0.032 (0.850) | 0.137 (0.329)* | 0.186 (0.098)* | 0.078 (0.294) | 0.015 (0.943) | -0.028 (0.199) | 0.152 (0.422) | 0.149 (0.435) | 0.116 (0.412)* | -0.057 (0.220) | 0.274 (0.078)* |
|  |  |  |  |  |  |  |  |  |  |  |  |  |  |  |  |  |
| *Gesture* | Events | Combined | Non-combined | Facial expression | Attention present | Attention absent | Penile erection | Objects | Non-objects | Repetitive | Non-repetitive | Close proximity | Far proximity | Piloerection | Repertoire size | Response present |
| Age | 0.173 (1.616)* | 0.109 (0.392) | 0.191 (1.302)* | 0.058 (0.131) | 0.119 (0.949) | 0.116 (0.558) | -0.020 (0.219) | 0.060 (0.743) | 0.187 (1.429)* | 0.159 (1.036)* | 0.147 (1.404) | 0.157 (0.726) ** | 0.117 (1.304) | 0.169 (1.307) | 0.122 (1.139) | 0.112 (0.729) |
| Sex | -0.022 (1.287) | -0.078 (0.342) | 0.002 (1.060) | 0.068 (0.108) | 0.042 (0.756) | 0.051 (0.458) | -0.176 (0.189)* | -0.069 (0.649) | 0.007 (1.131) | 0.004 (0.857) | -0.042 (1.157) | 0.067 (0.579) | -0.035 (1.085) | -0.070 (1.102) | -0.024 (0.948) | 0.019 (0.591) |
| Kinship | -0.031 (2.817) | -0.038 (0.704) | -0.017 (2.315) | 0.007 (0.239) | -0.007 (1.680) | 0.021 (1.029) | -0.074 (0.394) | -0.063 (1.319) | -0.007 (2.392) | -0.029 (1.850) | 0.014 (2.425) | 0.033 (1.268) | -0.050 (2.250) | -0.029 (2.256) | -0.038 (2.087) | 0.012 (1.341) |
| Reproductive status | 0.059 (1.288) | 0.067 (0.333) | 0.038 (1.073) | 0.112 (0.117) | 0.074 (0.860) | 0.021 (1.029) | 0.023 (0.180) | -0.038 (0.594) | 0.087 (1.325) | 0.003 (0.828) | 0.094 (1.194) | 0.092 (0.646) | -0.006 (1.051) | 0.032 (1.008) | 0.023 (0.956) | 0.083 (0.643) |
| Left-handed | 0.560 (0.374) ** | 0.207 (0.080)* | 0.619 (0.332)** | 0.303 (0.027)* | 0.327 (0.196)* | 0.692 (0.163) ** | -0.046 (0.049) | 0.192 (0.163)* | 0.577 (0.358) ** | 0.500 (0.229)** | 0.418 (0.266)* | 0.699 (0.209) ** | 0.317 (0.243)* | 0.349 (0.251)* | 0.515 (0.249)* | 0.683 (0.184) ** |
| Right-handed | 0.081 (1.130) | 0.148 (0.282) | 0.034 (0.900) | -0.087 (0.086) | 0.107 (0.687) | 0.041 (0.407) | 0.446 (0.178) ** | 0.076 (0.549) | 0.072 (1.020) | 0.135 (0.727) | 0.014 (0.905) | 0.038 (0.527) | -0.043 (0.856) | -0.082 (0.881) | -0.017 (0.835) | -0.107 (0.497)* |
|  |  |  |  |  |  |  |  |  |  |  |  |  |  |  |  |  |
| *Gesture* | Response absent | Repetition | Elaboration |  |  |  |  |  |  |  |  |  |  |  |  |  |
| Age | 0.179 (0.522)* | 0.115 (0.060) | -0.115 (0.116) |  |  |  |  |  |  |  |  |  |  |  |  |  |
| Sex | 0.049 (0.448) | 0.009 (0.051) | 0.087 (0.102) |  |  |  |  |  |  |  |  |  |  |  |  |  |
| Kinship | 0.007 (0.924) | -0.015 (0.107) | -0.055 (0.206) |  |  |  |  |  |  |  |  |  |  |  |  |  |
| Reproductive status | 0.060 (0.427) | -0.024 (0.044) | 0.040 (0.102) |  |  |  |  |  |  |  |  |  |  |  |  |  |
| Left-handed | 0.372 (0.110)* | -0.048 (0.011) | 0.030 (0.026) |  |  |  |  |  |  |  |  |  |  |  |  |  |
| Right-handed | 0.253 (0.357)* | 0.189 (0.046) | 0.253 (0.095)* |  |  |  |  |  |  |  |  |  |  |  |  |  |

* *p* < 0.05, ** *p* < 0.01, *** *p* < 0.001
