# Supplementary material for: Complex Sociality of Wild Chimpanzees Can Emerge from Laterality of Manual Gestures
Source: Hum Nat. 2019 Jun 24;30(3):299–325. doi: 10.1007/s12110-019-09347-3 (PMC6698263; doi:10.1007/s12110-019-09347-3)
Supplement: Supplementary file 8 — (DOCX 13 kb) [file 12110_2019_9347_MOESM6_ESM.docx]

Electronic Supplementary Material (ESM) - 6

for

Complex Sociality of Wild Chimpanzees Can Emerge from Laterality of Manual Gestures

Anna Ilona Roberts, Lindsay Murray, Sam George Bradley Roberts

*Human Nature* 30(3), 2019. Doi: to be added in proofs.

**Summary of results**

Multiple Regression Quadratic Assignment Procedure (MRQAP) regression models predicting rate of production of gestures per hour spent within 10 meters of the recipient of the gesture categorized according to function from rates of right-handed and left-handed gestures produced per hour spent within 10 meters. Summary table provides standardized coefficients (standard errors) and *p* values. Green shading indicates statistically significant positive relationships, red shading indicates statistically significant negative relationships. Full results are provided in Supplementary Tables.

| Gesture context | Left-handed gesture | Right-handed gesture |
| --- | --- | --- |
| Copulation | -0.156 (0.037)* | 0.505 (0.149)** |
| Threat to dominate | 0.396 (0.030)* | -0.235 (0.113)** |
| Gesture to groom give | 0.160 (0.089)* | 0.196 (0.318)* |
| Gesture to groom mutual | 0.406 (0.030)* | -0.237 (0.116)** |
| Gesture to groom receive | 0.439 (0.043)* | -0.096 (0.131) |
| Other threat | -0.119 (0.018)* | 0.187 (0.065)* |
| Greeting | 0.036 (0.043) | 0.167 (0.158) |
| Synchronized low intensity pant-hoot | -0.063 (0.016) | 0.107 (0.065) |
| Synchronized high intensity pant-hoot | 0.105 (0.042)* | -0.063 (0.182) |
| Solo high intensity pant-hoot | 0.222 (0.024)* | -0.098 (0.087)* |

* *p* < 0.05, ** *p* < 0.01, *** *p* < 0.001
